# A Type 2C Protein Phosphatase FgPtc3 Is Involved in Cell Wall Integrity, Lipid Metabolism, and Virulence in *Fusarium graminearum*

**DOI:** 10.1371/journal.pone.0025311

**Published:** 2011-09-28

**Authors:** Jinhua Jiang, Yingzi Yun, Qianqian Yang, Won-Bo Shim, Zhengyi Wang, Zhonghua Ma

**Affiliations:** 1 Key Laboratory of Molecular Biology of Crop Pathogens and Insects, Institute of Biotechnology, Zhejiang University, Hangzhou, China; 2 Department of Plant Pathology and Microbiology, Texas A&M University, College Station, Texas, United States of America; University of Wisconsin – Madison, United States of America

## Abstract

Type 2C protein phosphatases (PP2Cs) play important roles in regulating many biological processes in eukaryotes. Currently, little is known about functions of PP2Cs in filamentous fungi. The causal agent of wheat head blight, *Fusarium graminearum*, contains seven putative PP2C genes, *FgPTC1*, -*3*, -*5*, -*5R*, -*6*, -*7* and -*7R*. In order to investigate roles of these PP2Cs, we constructed deletion mutants for all seven PP2C genes in this study. The *FgPTC3* deletion mutant (ΔFgPtc3-8) exhibited reduced aerial hyphae formation and deoxynivalenol (DON) production, but increased production of conidia. The mutant showed increased resistance to osmotic stress and cell wall-damaging agents on potato dextrose agar plates. Pathogencity assays showed that ΔFgPtc3-8 is unable to infect flowering wheat head. All of the defects were restored when ΔFgPtc3-8 was complemented with the wild-type *FgPTC3* gene. Additionally, the *FgPTC3* partially rescued growth defect of a yeast *PTC1* deletion mutant under various stress conditions. Ultrastructural and histochemical analyses showed that conidia of ΔFgPtc3-8 contained an unusually high number of large lipid droplets. Furthermore, the mutant accumulated a higher basal level of glycerol than the wild-type progenitor. Quantitative real-time PCR assays showed that basal expression of *FgOS2*, *FgSLT2* and *FgMKK1* in the mutant was significantly higher than that in the wild-type strain. Serial analysis of gene expression in ΔFgPtc3-8 revealed that *FgPTC3* is associated with various metabolic pathways. In contrast to the *FgPTC3* mutant, the deletion mutants of *FgPTC1*, *FgPTC5*, *FgPTC5R*, *FgPTC6*, *FgPTC7* or *FgPTC7R* did not show aberrant phenotypic features when grown on PDA medium or inoculated on wheat head. These results indicate FgPtc3 is the key PP2C that plays a critical role in a variety of cellular and biological functions, including cell wall integrity, lipid and secondary metabolisms, and virulence in *F. graminearum*.

## Introduction

Reversible phosphorylation of proteins controlled by protein kinases and phosphatases is an important mechanism for regulating numerous biological processes in eukaryotes. Protein phosphorylation and dephosphorylation generally occurs at tyrosine, serine, or threonine residues. Based on the substrate specificity, protein phosphatases are classed in two major groups: serine/threonine (Ser/Thr) phosphatases and tyrosine phosphatases (PTPs) [1]. Ser/Thr protein phosphatases have been classically categorized into two superfamilies: PPPs (phosphoprotein phosphatases) and PPMs (metal-dependent protein phosphatases). The PPP family consists of PP1, PP2A, and PP2B phosphatases. The PPM family contains type 2C protein phosphatases (PP2Cs) and pyruvate dehydrogenase phosphatase. PP2Cs are normally monomeric enzymes and require metal cation Mg^2+^ or Mn^2+^ for their dephosphorylation activities [2]–[5].

PP2Cs have a variety of cellular functions in both prokaryotes and eukaryotes. In *Salmonella enterica*, PrpZ, a PP2C, is involved in virulence. The deletion mutant of prpZ gene cluster showed a significantly lower level of survival than the wild-type progenitor in human macrophages [6]. In mammalian cells, at least 16 distinct PP2Cs have been identified, and they are involved in the regulation of various cellular functions including response to stress, cell cycle regulation, actin cytoskeleton organization, and pre-mRNA splicing [1], [7]. *Arabidopsis* contains an unusually large group of more than 80 PP2C proteins, which are involved in the regulation of diverse signaling pathways [8], [9]. The PP2Cs ABI1 and ABI2 have been shown as major negative regulators of ABA signaling by interacting physically with SNF1-related protein kinases [10]. In *Saccharomyces cerevisiae*, seven PP2C phosphatase genes (*PTC1∼PTC7*) have been identified. Ptc1, Ptc2, and Ptc3 have been shown to negatively regulate the high-osmolarity glycerol (HOG) pathway [11]–[14]. Ptc1 performs its functions on the HOG pathway through the adaptor protein Nbp2, whose N-terminal domain and the SH3 domain are necessary for interaction with Ptc1 and binding to Pbs2, respectively [14]. In addition to inactivating Hog1, these PP2Cs also perform many other biological functions. Ptc1 is involved in the regulation of cell wall integrity (CWI), tRNA splicing, sporulation, lithium tolerance [15], [16]. It is also required for the temporal control of distributing mitochondria, cortical ER, and vacuole [17], [18]. Both Ptc2 and Ptc3 are responsible for the dephosphorylation of cyclin-dependent kinases (CDK) [19]. Furthermore, phosphorylated Ptc2 and Ptc3 can dephosphorylate the checkpoint protein kinase Rad53, which is necessary for the adaptation to double-strand break of DNA induced by HO endonuclease [20], [21]. In *Schizosaccharomyces pombe*, Ptc4 regulates vacuole fusion by dephosphorylating one or more proteins in vacuole membrane. The cells of Ptc4 deletion mutant are unable to fuse vacuoles in response to hypotonic stress or nutrient starvation. Conversely, Ptc4 overexpression leads to stimulated vacuole fusion [4], [5], [22].

Compared to Ptc1, little is known about the functions of other three PP2C phosphatases (Ptc5, Ptc6 and Ptc7) in *S. cerevisiae*. Disruption of *PTC5* gene (ORF *YGL059*) had little effect on yeast growth on glucose, but caused retardation on acetate. Growth rate of the mutant was restored to the wild-type level by simultaneous disruption of the *PDA1* gene, which encodes the *α* subunit of pyruvate dehydrogenase complex [23]. A further study showed that both Ptc5 and Ptc6 (*YCR079*) are involved in the regulation of this complex by affecting the phosphorylation state of Pda1 [24]. Tal *et al.* [25] found that Ptc6 is required for efficient survival of yeast cells in prolonged stationary phase cultures. Additionally, Ptc6 is also associated with the sensitivity to rapamycin in *S. cerevisiae*. The *PTC6* deletion mutant showed increased sensitivity to rapamycin, whereas overexpression of *PTC6* caused yeast cells to be resistant to rapamycin [26]. Although *PTC7* deletion mutants did not exhibit any morphological changes, overexpression of the gene conferred a growth advantage on 2% ethanol or 2% galactose medium in a low O_2_ growth environment. This suggested a possible role of *PTC7* in the utilization of nonfermentable carbon sources in low O_2_ growth environments [27].

Genome-wide search for PP2C in the filamentous fungi, including *Aspergullus* species, *Fusarium graminearum*, *Neurospora crassa*, and *Magnaporthe oryzae*, revealed that all these genomes harbor multiple putative PP2C proteins [5]. Analysis of the *F. graminearum* genome database (available at http://www.broadinstitute.org/annotation/genome/fusarium_group/MultiHome.html) revealed that the fungus contains seven putative genes encoding PP2Cs. However, to date, our understanding of functions of PP2C in filamentous fungi is very limited. The sole study on function of PP2C in filamentous fungi was conducted by Jiang *et al.* [28], and the authors found that the deletion of *F. graminearum* PP2C gene *FgPTC1* results in increased sensitivity slightly to LiCl during mycelial growth. The mutant also showed reduced virulence on wheat coleoptile, suggesting that FgPtc1 plays an role in regulating hyphal growth and virulence in *F. graminearum* [28].

*F. graminearum* (teleomorph *Gibberella zeae*) causes Fusarium head blight (FHB), which is a devastating disease of cereal crops worldwide [29]. While yield loss caused by the disease is a major concern, mycotoxins produced by the fungus in infected grains pose a serious threat to human and animal health. Currently, most wheat cultivars planted in the world are susceptible to *F. graminearum*; therefore, the primary method for management of FHB is through the application of fungicides during wheat anthesis. To date, only few fungicides (including benzimidazoles, triazoles, and carboximides) have been registered for the control of FHB, and they normally provide only approximately 50% reduction of FHB index and 40% reduction in deoxynivalenol [30]. Additionally, *F. graminearum* has developed resistance to some of the fungicides [31]. Therefore, the development of new fungicides is desperately needed for effective management of FHB and mycotoxin contamination in cereals. Considering how important PP2Cs are for fungal viability and pathogenicity, they can be identified as attractive antimicrobial targets for therapeutic purposes. With this aim, we characterized PP2Cs in *F. graminearum*, which may help in exploitation of drug targets for the design of new anti-mycotoxin and antifungal agents.

## Results

### Sequence analysis of FgPP2Cs

Search for PP2C homologs in *F. graminearum* genome showed that this fungus contains seven putative PP2C genes: *FgPTC1*, -*3*, -*5*, -*5R* (*FgPTC5*-related family), -*6*, -*7* and -*7R* (*FgPTC7*-related family). The primary structures of these genes are presented in Table 1. Reverse transcription PCR analyses showed that each of these seven genes was expressed in *F. graminearum* mycelia grown on PDA medium (data not shown). The predicted amino acid sequences of *F. graminearum* PP2Cs share substantial identity (24–43%) to those of *S. cerevisiae* PP2C members (Table 1, Figure S2A). CDART (conserved domain architecture retrieval tool) program [32] predicted that all seven proteins harbor conserved PP2C Ser/Thr phosphatases domain (Figure S2B).

**Table 1:** The primary structures of PP2C genes from *F. graminearum*.

Gene	Length of nucleotide sequence (bp)	No. of intron	No. of predicted amino acid	Percentage of amino acid identical to *S. cerevisiae* PP2C members
*FgPTC1*	1,781	1	577	43% to Ptc1
*FgPTC3*	2,049	6	430	42% to Ptc3
*FgPTC5*	1,902	2	594	38% to Ptc5
*FgPTC5R*	1,497	1	482	33% to Ptc5
*FgPTC6*	2,004	0	667	36% to Ptc6
*FgPTC7*	1,644	1	394	30% to Ptc7
*FgPTC7R*	1,253	2	381	24% to Ptc7

### Disruption of PP2Cs in *F. graminearum*

To investigate the role of FgPP2Cs, we generated single gene deletion mutants of *FgPTC1*, -*3*, -*5*, -*5R*, -*6*, -*7* and -*7R* using a homologous recombination strategy. For *FgPTC1*, 15 deletion mutants were identified from 20 hygromycin-resistant transformants by PCR analysis with the primer pair P15+P16 (Table S1). A deletion mutant ΔFgPtc1-5 was further verified through Southern analysis. When probed with a 981-bp upstream DNA fragment of *FgPTC1* (Figure S1A), ΔFgPtc1-5 had an anticipated 3,510-bp band, but lacked the 6,156-bp band which was present in the wild-type PH-1 (Figure S1B). This Southern hybridization pattern confirmed that ΔFgPtc1-5 is a null mutant resulting from a single homologous recombination event at the *FgPTC1* locus.

For *FgPTC3* gene, 10 deletion mutants were identified from 15 hygromycin-resistant transformants by PCR analysis with primer pair P35+P36 (Table S1). When performed Southern analysis with a 977-bp downstream DNA fragment of *FgPTC3* as a probe (Figure S3A), we found that the deletion mutant ΔFgPtc3-8 had the expected 3,020-bp band while the wild-type progenitor had a 3,668-bp band (Figure S3B). To confirm that phenotypic changes observed in ΔFgPtc3-8 were due to the deletion of *FgPTC3* gene, the mutant was complemented with a full-length wild-type *FgPTC3* gene. Southern analysis confirmed the insertion of a 5,459-bp fragment in the complemented strain ΔFgPtc3-8C (Figure S3B). PCR analyses showed that primer pairs hph-F + neo-R and hph-F + P32 (Table S1) generated a 2,619-bp and 4,534-bp amplicon, respectively, from the complemented strain. These results indicated that *FgPTC3* gene was re-introduced to the original *FgPTC3* locus in complemented strain ΔFgPtc3-8C.

To date, *S. cerevisiae* Ptc1 is the best characterized PP2C, and it plays specific functional roles that are not shared by other PP2C members [5]. However, the deletion mutant of *FgPTC1* did not show observable phenotypic changes as compared to the wild type progenitor (see results below). Thus, we were interested in generating a double mutant of *FgPTC1* and *FgPTC3* to see whether or not there is a synergetic effect between two genes. The *FgPTC1/FgPTC3* double mutant ΔFgPtc13-6 was obtained by transforming ΔFgPtc3-8 mutant with the disruption vector pCA-FgPtc13-Del, and subsequently verified by PCR analysis with the primer pair P15+P16. Southern analysis with a 981-bp upstream DNA fragment of *FgPTC1* as the probe showed that *FgPTC1* gene was successfully deleted and thus resulting in the double mutant ΔFgPtc13-6 (Figure S1B).

For the remaining five PP2C genes, we obtained nine *FgPTC5*, six *FgPTC5R*, twelve *FgPTC6*, seven *FgPTC7* and ten *FgPTC7R* deletion mutants identified by PCR assays. Southern hybridization confirmed that the mutants FgPtc5-5, FgPtc5R-2, FgPtc6-9, FgPtc7-3 and FgPtc7R-2 resulted from a single homologous recombination event at the respective gene locus (data not shown).

In *S. cerevisiae*, Ptc1 is involved in HOG pathway by dephosphorylating the Hog1 mitogen-activated protein (MAP) kinase [14]. In order to investigate the relationships between PP2Cs and HOG pathway, we also generated the deletion mutant of *FgOS2*, which is a homolog of *S. cerevisiae Hog1*. A previous study has showed that FgOs2 is a key element of the HOG pathway in *F. graminearum*. The *FgOS2* deletion mutant showed increased sensitivity dramatically to osmotic stress [33]. By comparing phenotypes of *F. graminearum* PP2C mutants against those of *FgOS2* mutant, we could obtain some genetic evidences on whether or not PP2Cs are involved in the HOG pathway in *F. graminearum*.

### Involvement of FgPtc3 in the regulation of vegetative growth and pigment formation

**Figure 1:**
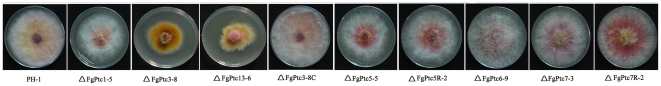
Impact of *FgPTC3* on colony morphology and pigment formation. The wild-type strain PH-1, the mutant ΔFgPtc1-5, ΔFgPtc3-8, ΔFgPtc13-6, ΔFgPtc3-8C, ΔFgPtc5-5, ΔFgPtc5R-2, ΔFgPtc6-9, ΔFgPtc7-3 and ΔFgPtc7R-2 were grown on PDA for 4 days at 25°C.

The *FgPTC3* deletion mutant ΔFgPtc3-8 grew much slower than the wild-type progenitor PH-1 and six other PP2C mutants on PDA (Figure 1) and MM medium (data not shown). Additionally, ΔFgPtc3-8 exhibited a reduced aerial hyphae formation, but increased production of yellow pigment (Figure 1). To further confirm the finding that *FgPTC3* affects pigment biosynthesis, we assayed the expression of *PKS12* gene, which encodes a type I polyketide synthase necessary for pigment biosynthesis. Quantitative real-time PCR (qRT-PCR) analysis showed that the expression level of *PKS12* in ΔFgPtc3-8 was increased by 3.2-fold in comparison to that in PH-1. The double mutant of *FgPTC1* and *FgPTC3*, ΔFgPtc13-6, displayed similar morphological changes as those observed in ΔFgPtc3-8 (Figure 1), indicating that the deletion of *FgPTC1* had not additive effects on morphological characters of ΔFgPtc3-8. These results indicate that FgPtc3 plays a prominent role in the regulation of vegetative growth and pigment formation in *F. graminearum*.

Compared to the wild-type strain, the deletion mutants of six other PP2Cs (*FgPTC1*, -*5*, -*5R*, -*6*, -*7* and -*7R*) did not show observable phenotypic changes in mycelial growth on PDA (Figure 1) and virulence on wheat head (Figure S4), and thus they were not further used in the following experiments.

### Involvement of FgPtc3 in the regulation of conidiation and lipid metabolism

**Figure 2:**
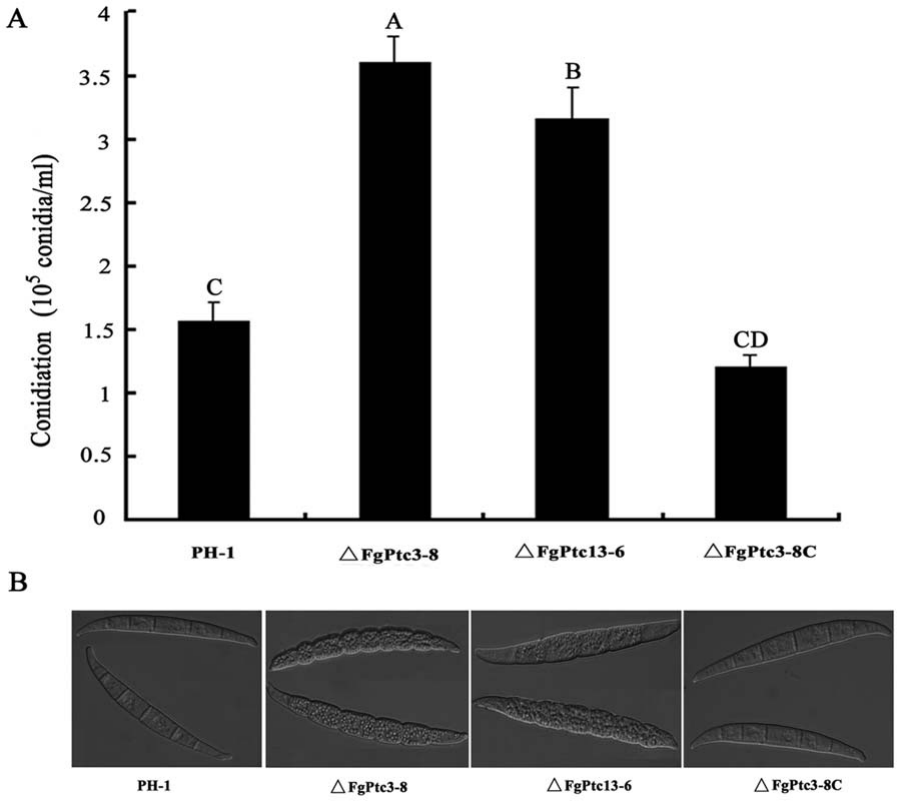
Impact of *FgPTC3* on conidiation of *F. graminearum*. (A) Conidia were accounted after incubation of the wild-type strain PH-1, the mutant ΔFgPtc3-8, ΔFgPtc13-6, or ΔFgPtc3-8C in mung bean liquid medium for 4 days. Blank bars with the same letter are not significantly different at *P* = 0.05 (ANOVA of SAS). (B) Differential interference contrast [DIC] images of conidia were captured with an electronic microscope.

**Figure 3:**
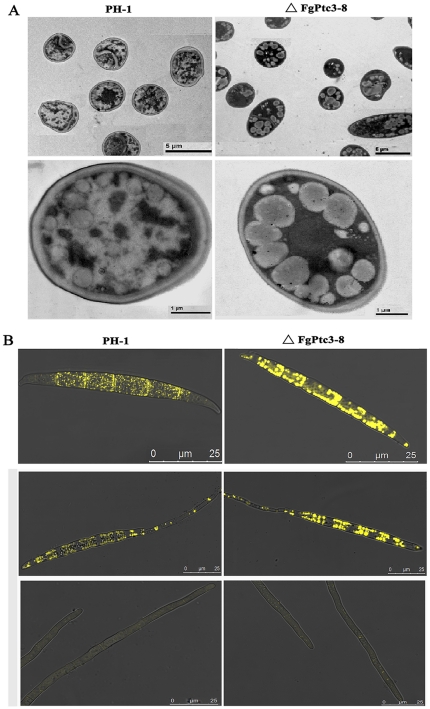
Ultrastructural and histochemical analyses of lipid droplets within conidia of mutant ΔFgPtc3-8. (A) Lipid drops within conidia of the wild type PH-1 and the mutant **Δ**FgPtc3-8 examined with a transmission electronic microscope. (B) Lipid drops in conidium [top] and germinating conidium [middle] and hyphae [bottom] were stained with Nile Red and examined under a microscope with episcopic fluorescence.

When grown in MBL medium, the *FgPTC3* mutant (ΔFgPtc3-8) and *FgPTC1* and -*3* double mutant (ΔFgPtc13-6) produced more conidia than the wild-type progenitor PH-1 (Figure 2A). Microscopic observation showed that conidia of ΔFgPtc3-8 and ΔFgPtc13-6 mutants contain many subcellular particles that were not typically seen in PH-1 (Figure 2B). To characterize these particles in detail, we examined the conidia using transmission electron microscopy. Numerous large lipid droplets were observed in ΔFgPtc3-8 conidia, but few were observed in PH-1 (Figure 3A). The lipid droplets were further verified by histochemical staining with Nile Red. As shown in Figure 3B, numerous large discrete lipid droplets were highlighted in the ungerminating and germinating conidia of ΔFgPtc3-8, but only few were detected in the wild-type strain. Interestingly, the lipid droplets were almost completely degraded in hyphae of the mutant. These results strongly indicate that *FgPTC3* is involved in the regulation of lipid metabolism in *F. graminearum*.

### Requirement of FgPtc3 in virulence of *F. graminearum*

**Figure 4:**
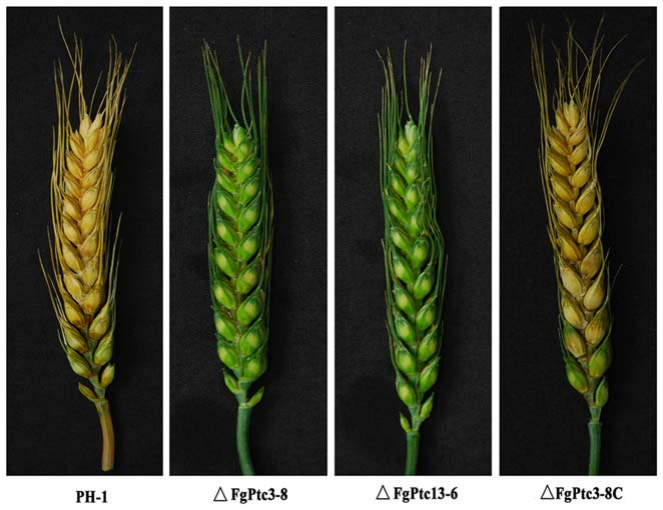
Virulence of PH-1, the mutant ΔFgPtc3-8, ΔFgPtc13-6 and ΔFgPtc3-8C on wheat heads. Wheat heads were point-inoculated with conidial suspension of each strain, and infected wheat heads were examined 15 days after inoculation.

Virulence of ΔFgPtc3-8 was evaluated by point inoculating conidial suspension on wheat head. Fifteen days after inoculation, scab symptoms failed to develop in the spikelets point-inoculated with ΔFgPtc3-8 and ΔFgPtc13-6 (Figure 4). Under the same conditions, however, scab symptoms developed in more than 90% spikelets when wheat heads were point-inoculated with the wild-type PH-1 or the complemented strain ΔFgPtc3-8C (Figure 4).

### Role of FgPtc3 in the regulation of deoxynivalenol (DON) biosynthesis

**Figure 5:**
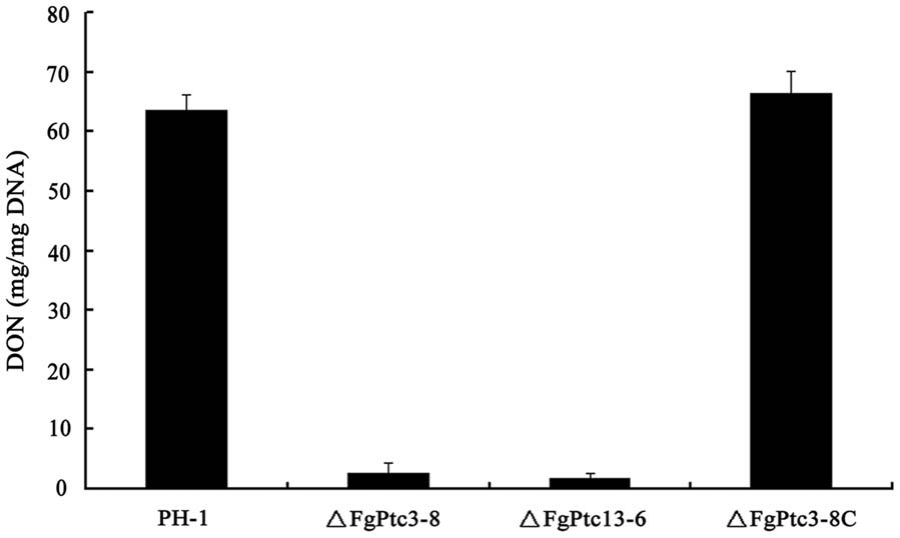
Effects of FgPtc3 on the biosynthesis of DON. Amount of DON (per mg fungal DNA) produced by the wild type PH-1, the mutants ΔFgPtc3-8, ΔFgPtc13-6, or ΔFgPtc3-8C were detected in infected wheat kernels after 20 days of incubation. Line bars in each column denote standard errors of three experiments.

DON is an important virulence factor in *F. graminearum* [34]–[36]. Since ΔFgPtc3-8 was unable to infect flowering wheat head, we analyzed the level of DON produced in the gene deletion mutant. When cultured on wheat kernels for 20 days, the levels of DON produced by the wild-type strain were 30 times higher than that produced by ΔFgPtc3-8 (Fig. 5). Similar to ΔFgPtc3-8, the double mutant ΔFgPtc13-6 produced a very low level of DON on wheat kernels. Complementation of *FgPTC3* restored the ability of ΔFgPtc3-8 to produce DON at a wild type level (Figure 5).

To further confirm the finding that FgPtc3 affects DON biosynthesis, we assayed the expression of *TRI5* gene encoding trichodiene synthase by quantitative real-time PCR (qRT-PCR) using RNA samples isolated from mycelia grown in GYEP medium for 2 days. The expression level of *TRI5* in the mutant ΔFgPtc3-8 was diminished by 2.5 folds compared to that of PH-1 (data not shown). Thus, we concluded that FgPtc3 is necessary for proper expression of *TRI5* and subsequent DON biosynthesis in *F. graminearum*.

### The *FgPTC3* mutant is resistant to osmotic and metal cation stresses

**Figure 6:**
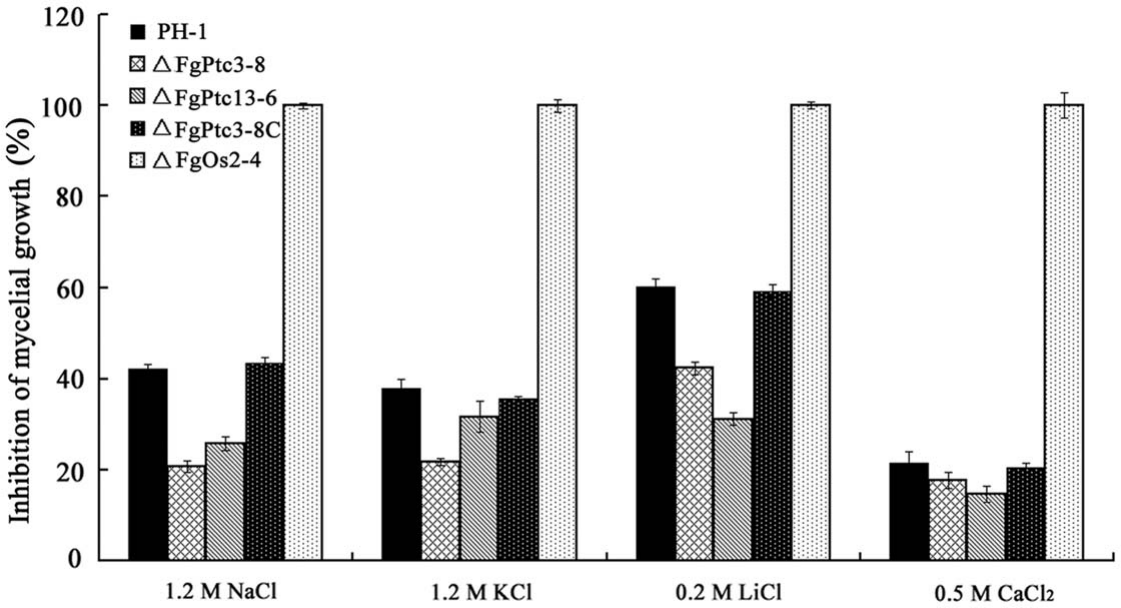
Sensitivity to osmotic stresses and metal cations of PH-1, ΔFgPtc3-8, ΔFgPtc13-6, ΔFgPtc3-8C, and ΔFgOs2-4. (A) Osmotic stresses were mediated by addition of NaCl or KCl in potato dextrose agar [PDA] medium. (B) Metal cations were added into minimal medium [MM]. Bars denote standard errors from three repeated experiments.

**Figure 7:**
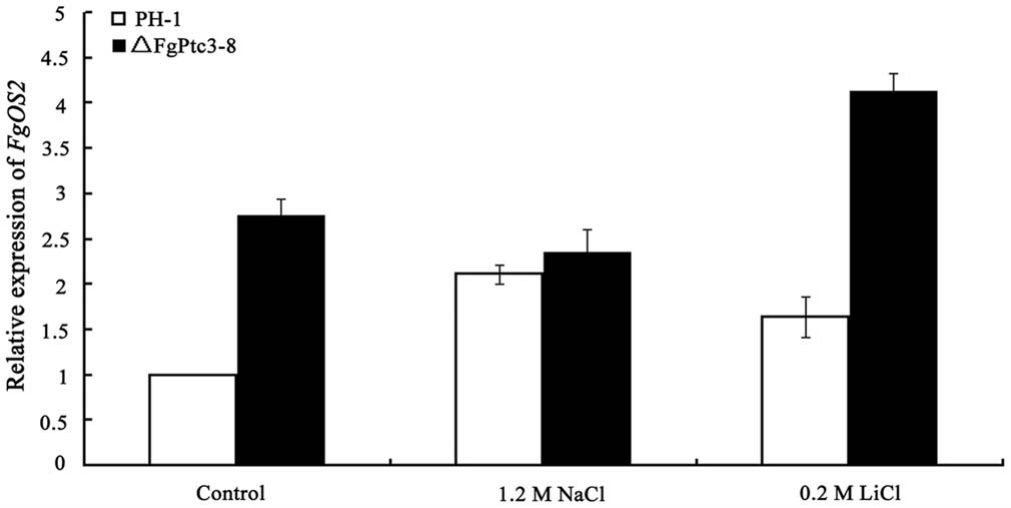
Relative expression levels of *FgOS2* in the wild-type strain PH-1 and the mutant ΔFgPtc3-8. RNA samples were extracted from mycelia of each strain untreated or treated with 1.2 M NaCl or 0.2 M LiCl for 2 hours after grown in potato dextrose broth for 2 days. The relative expression of *FgOS2* in the wild-type strain or ΔFgPtc3-8 is the relative amount of mRNA of each gene in the wild-type strain without treatment. Line bars in each column denote standard errors of three experiments.

In *S. cerevisiae*, one of the major roles of PP2C is to negatively regulate the HOG pathway. Thus, we compared ΔFgPtc3-8 against the *FgOS2* mutant ΔFgOs2-4 in their ability to cope with osmotic stress. As shown in Figure 6, ΔFgPtc3-8 and ΔFgPtc13-6 showed increased resistance to osmotic stress mediated by 1.2 M NaCl or 1.2 M KCl. In contrast, ΔFgOs2-4 was extremely sensitive to osmotic stress. The strain was virtually unable to grow on PDA amended with 1.2 M NaCl or 1.2 M KCl. Similar to osmotic sensitivity patterns, we found that ΔFgPtc3-8 exhibited increased resistance to LiCl, while ΔFgOs2-4 was very sensitive to LiCl and CaCl_2_ (Figure 6). These results suggest the possibility that FgPtc3 negatively regulates FgOs2 in *F. graminearum*. To further confirm this inference, we analyzed the expression of *FgOS2* in ΔFgPtc3-8. As shown in Figure 7, the basal expression of *FgOS2* in ΔFgPtc3-8 was significantly higher than that in the wild-type strain without osmotic stress. The expression of *FgOS2* gene was upregulated by high salt treatment in the wild-type strain, but not in ΔFgPtc3-8 (Figure 7). These results indicate that FgPtc3 could negatively regulate FgOs2 at transcription level. Additionally, to get further evidences at a level of protein, we tried to analyze phosphorylation profiles of FgOs2 using a Western blot approach. Unfortunately, a dually phosphorylated p38 (Thr180/Tyr182) antibody and Hog1 antibody correspondingly phosphorylated Hog1 and Hog1 in *S. cerevisiae* did not work well with *F. graminearum*.

**Figure 8:**
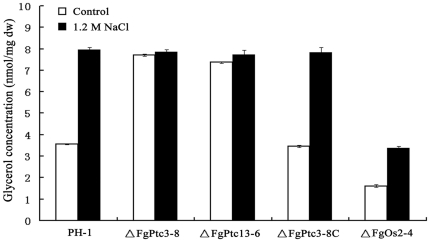
Effects of FgPtc3 and FgOs2 on the glycerol biosynthesis. Intracellular glycerol concentration [nmol/mg dried mycelia] in mycelia of the wild-type strain PH-1, and the mutant ΔFgPtc3-8, ΔFgPtc13-6, ΔFgPtc3-8C and ΔFgOs2-4 were analyzed after 2 h of 1.2 M NaCl treatment. Bars denote standard errors from three repeated experiments.

It has been reported that osmotic stress can induce glycerol accumulation in fungi via the HOG-like pathway [37], [38]. We therefore analyzed glycerol accumulation in mycelia of the mutant ΔFgPtc3-8 after 2 h of salt treatment. As shown in Figure 8, similar to the expression pattern of *FgOS2*, a basal level of glycerol concentration in ΔFgPtc3-8 was significantly higher than that in the wild-type strain and the *FgOS2* mutant (ΔFgOs2-4). The osmotic stress induced glycerol accumulation in wild-type strain and ΔFgOs2-4, but not in ΔFgPtc3-8. Together with *FgOS2* expression data, these results provide genetic evidences to confirm that FgPtc3 may negatively regulate HOG pathway in *F. graminearum*.

In *S. cerevisiae*, Ptc1 negatively regulates the HOG pathway through the adaptor protein Nbp2, whose N-terminal domain interact with Ptc1, and the SH3 domain is responsible for binding to Pbs2 [14]. Search for *F. graminearum* genome showed that this fungus has a homolog of *NBP2* gene, named *FgNBP2* (genome database accession No. FGSG_00929). We therefore analyzed interaction among FgPtc3, FgNpb2, and FgOs2. Yeast two-hybrid assays, however, showed that there was no interaction among FgPtc3, FgNbp2 and FgOs2 (Figure S5). Using *F. graminearum* protein-protein interaction (FPPI) program at http://csb.shu.edu.cn/fppi [39], FgPtc3 was predicted to interact with FgOs5 (a homolog of *S. cerevisiae* Pbs2). Unfortunately, the putative interaction between FgPtc3 and FgOs5 was not observed in our yeast two-hybrid assays (data not shown).

### The *FgPTC3* mutant is resistant to cell wall damaging agents

**Figure 9:**
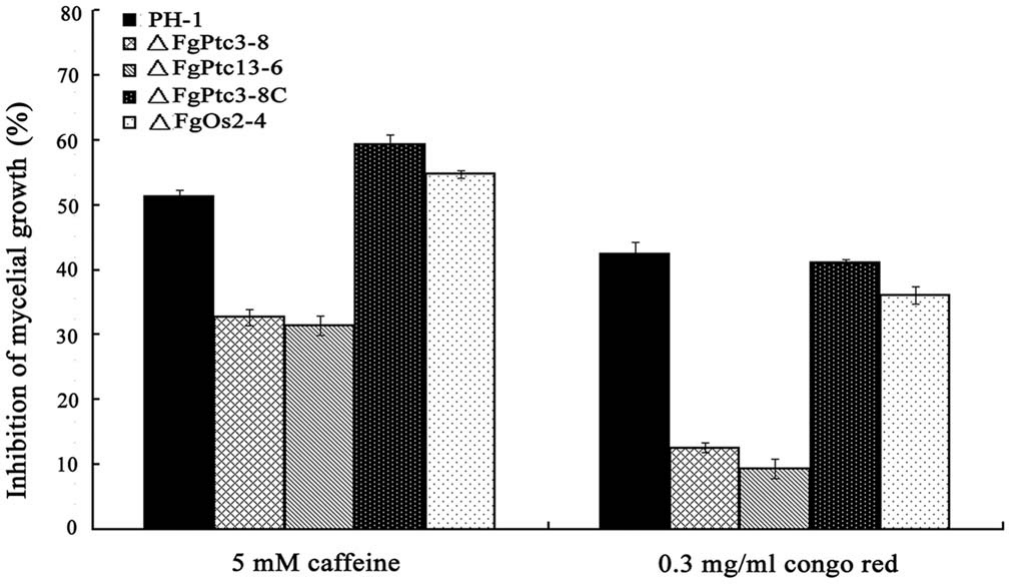
Sensitivity of PH-1, the mutant ΔFgPtc3-8, ΔFgPtc13-6, ΔFgPtc3-8C, and ΔFgOs2-4 to cell wall-damaging agents. Cell wall-damaging agents caffeine and congo red were added into potato dextrose agar medium. Bars denote standard errors from three repeated experiments.

The fluorochrome dyes calcoflour white and congo red can damage fungal cell wall by binding to chitin and cellulose [40]. When examined sensitivity of the mutants to these cell wall damaging agents, we observed that ΔFgPtc3-8 displayed increased tolerance to these compounds. Compared to the wild-type strain, however, ΔFgOs2-4 did not change its sensitivity significantly to caffeine and congo red (Figure 9). These results indicate that FgPtc3 may be involved in regulating cell wall integrity (CWI) pathway, which is independent from the HOG pathway.

**Figure 10:**
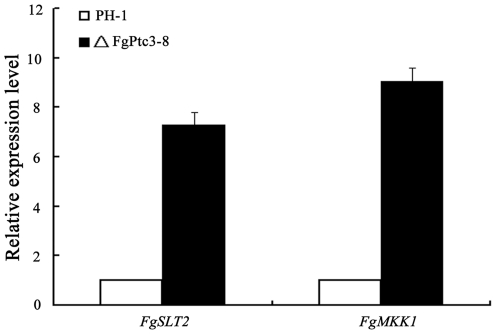
Relative expression levels of *FgSLT1* and *FgMKK1* in PH-1 and mutant ΔFgPtc3-8. RNA samples were extracted from mycelia of each strain after grown in potato dextrose broth for 2 days. The relative expression of *FgSLT1* and *FgMKK1* in ΔFgPtc3-8 is the relative amount of mRNA of each gene in the wild-type strain. Line bars in each column denote standard errors of three experiments.

To further confirm the involvement of FgPtc3 in the regulation of CWI pathway, we determined the expressions of *FgMKK1* (FGSG_07295) and *FgSLT?* (FGSG_10313), which are homologous to the *S. cerevisiae* CWI core element genes, *Mkk1/2* and *Slt2*, respectively. As shown in Figure 10, expression levels of *FgMKK1* and *FgSLT2* in ΔFgPtc3-8 were 9 and 7.2 folds higher than those in the wild-type strain. These results suggest that FgPtc3 negatively regulates transcriptions of some genes in the CWI pathway.

### FgPtc3 partially complemented the growth defect of yeast *PTC1* deletion mutant under various stress conditions

**Figure 11:**
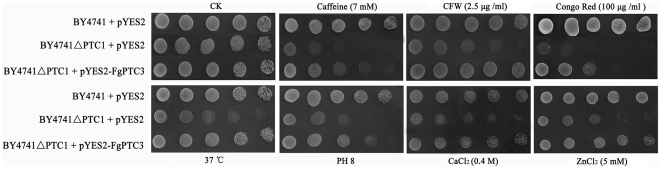
*F. graminearum* FgPtc3 complements the *S. cerevisiae PTC1* mutant. The yeast *PTC1* mutant was complemented with *FgPTC3* to generate the strain BY4741ΔPTC1 + pYES2-FgPTC3. The wild type strain BY4741 and *PTC1* mutant BY4741ΔPTC1 transformed with empty pYES2 vector were used as controls. Different dilutions of cell suspension of each strain were spotted on SG plates under different stresses. After incubated at 30°C [or 37°C as indicated] for four days, growth of each strain on each plate was examined.

In *S. cerevisiae*, *PTC1* deletion mutant, but not *PTC3* mutant, showed clear phenotypic changes under various stress conditions [5], [41]. In order to further determine functions of FgPtc3, we tested whether or not FgPtc3 would complement the yeast *PTC1* mutant. An expression vector pYES2 containing the full-length *FgPTC3* cDNA was transformed into the *PTC1* mutant BY4741ΔPTC1. As a control, the mutant was also transformed with an empty pYES2 vector. As shown in Figure 11, the yeast mutant and the wild type strain both grow well on SG medium without addition of any stress agents. However, the growth of *PTC1* mutant was significantly hindered when the medium was supplied with 7 mM caffeine, 2.5 µg/ml calcofluor white (CFW), 100 µg/ml Congo red, 0.4 M CaCl_2_, 5 mM ZnCl_2_. Additionally, the growth of *PTC1* mutant was also obstructed at a high pH (8.0) or at 37°C. The growth defects were partially restored by genetic complementation of yeast BY4741ΔPTC1 mutant with *F. graminearum FgPTC3* (Figure 11).

### Serial analysis of gene expression (SAGE) reveals that FgPtc3 is associated with various metabolism pathways

To further elucidate the function of FgPtc3 as well as identify downstream genes that it may impact, SAGE experiment was performed. After removal of low quality (<3) tags, we obtained a total of 125,406 and 134,754 distinct tags for PH-1 and ΔFgPtc3-8, respectively. Among these distinct tags, 70.02% and 77.48% can be uniquely mapped to the reference sequences for PH-1 and ΔFgPtc3-8, respectively. For SAGE data, the analysis is usually limited to a predefined tag showing at least 5-fold difference in abundance at a *P* value ≤0.05 [42] . With this criterion, we identified 1,369 genes up-regulated (>5-folds) and 410 genes down-regulated (<0.2-folds) in ΔFgPtc3-8 compared to PH-1.

**Figure 12:**
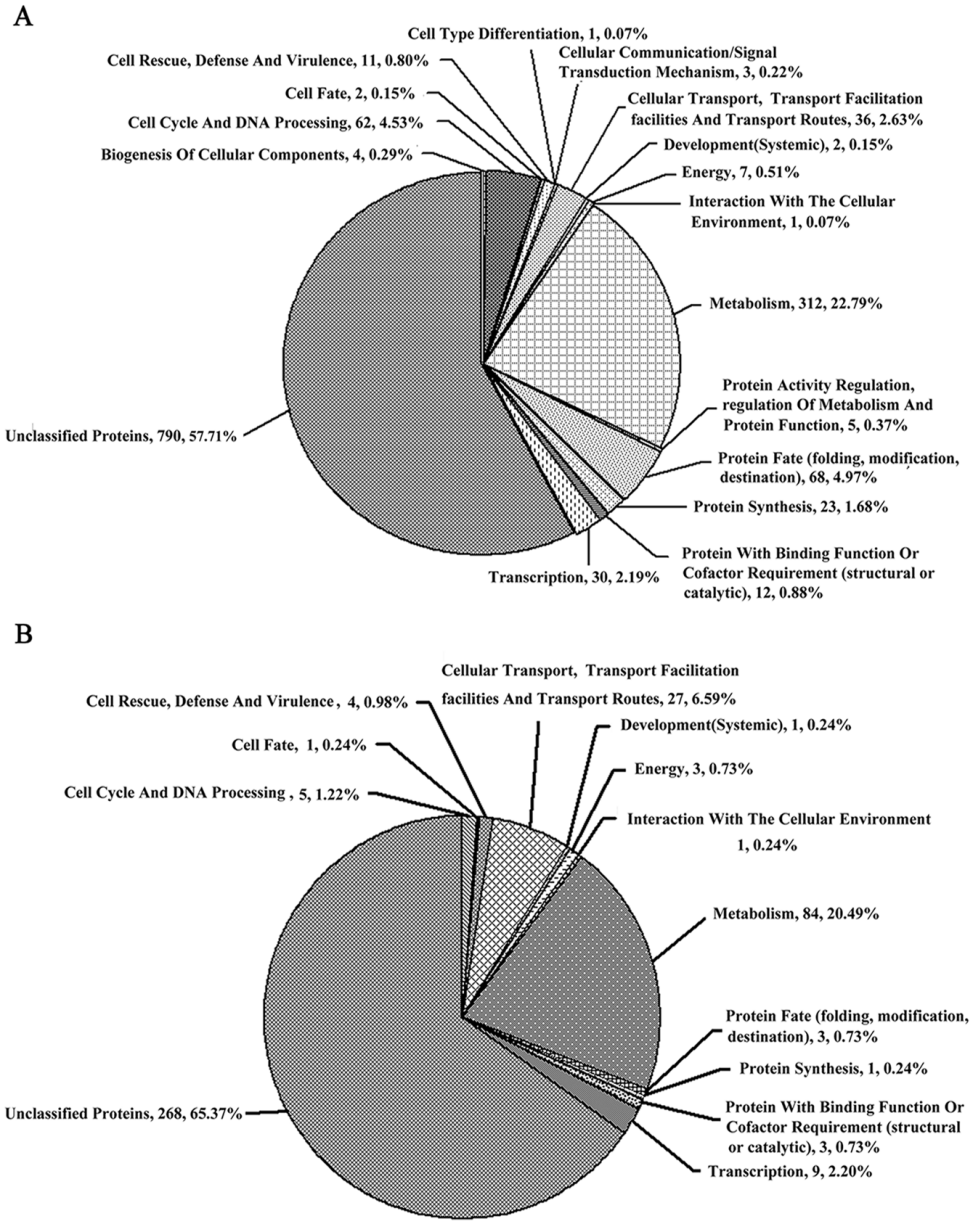
Pie chart grouping the genes up- and down-regulated in expression in ΔFgPtc3-8 compared with PH-1. (**A**) 1369 genes up-regulated more than 5 folds in the mutant ΔFgPtc3-8 compared with wild-type PH-1. (**B**) 410 genes down-regulated more than 5 folds in the mutant ΔFgPtc3-8 compared with wild-type PH-1. The expressions of genes were detected by the serial analysis of gene expression method.

To obtain better understanding of the overall gene expression profile, the upregulated and downregulated genes were grouped into several functional categories using FunCat program (http://mips.helmholtz-muenchen.de/proj/funcatDB/search_main_frame.html). Among the 1,369 genes upregulated in ΔFgPtc3-8, 312 (22.79%) grouped into the functional category of metabolism, comprising the largest group apart from the unclassified genes (Figure 12A). Among the 410 downregulated genes, again, 84 (20.49%) associated with various metabolisms to comprise the largest group, except for the unclassified 268 genes (Figure 12B). These results suggest that FgPtc3 is associated with various metabolism pathways.

Since numerous lipid droplets were observed in ΔFgPtc3-8 conidia, we also paid attention on expressions of the genes involved in fatty acid biosynthesis and metabolism. As shown in Table S2, among 40 genes, 28 and 3 genes involved in fatty acid metabolism were up- and down-regulated more than five folds, respectively. In addition to fatty acid synthesis and metabolism, expressions of genes, which are associated with several other metabolisms, were changed dramatically in the *FgPTC3* mutant (Figure S6). These results further support that FgPtc3 is involved in various cellular processes.

## Discussion

In an earlier study, Jiang *et al.* [28] found that the deletion of *FgPTC1* attenuates the virulence of *F. graminearum* on wheat coleoptiles. But the authors did not test virulence of the mutant on wheat head. In current study, when inoculated on wheat head, we found that ΔFgPtc1-5 did not exhibit decreased virulence (Figure S4). Additionally, *FgPTC5*, -*5R*, -*6*, -*7* and -*7R* deletion mutants retain high virulence on wheat head (Figure S4). In contrast, ΔFgPtc3-8 completely lost virulence. Additionally, ΔFgPtc3-8 grew significantly slower, but produced more conidia than wild type progenitor PH-1. These results indicate that FgPtc3 plays more important role than FgPtc1, -5, -5R, -6, -7 and -7R in regulating vegetative differentiations and virulence in *F. graminearum*.

DON is an end product of the trichothecene biosynthetic pathway and it has been identified as an important virulence factor in *F. graminearum* [34]–[36]. A deletion of *TRI5* gene, which encodes the enzyme trichodiene synthase to catalyze the first step in trichothecene biosynthesis, led to reduced virulence of *F. graminearum* on wheat head. When further investigated the role of DON in pathogenesis, Bai *et al.* [43] found that DON plays a significant role in the spread of FHB within a spike, but is not necessary for fungal initial infection. In our study, ΔFgPtc3-8 revealed a significant reduction in DON production, which is consistent with the observation that ΔFgPtc3-8 was compromised in its ability to infect plant host. In contrast to DON biosynthesis, we found that the *FgPTC3* mutant displayed increased biosynthesis of the yellow pigment. These results indicate that a PP2C protein can simultaneously act as positive and negative regulator of secondary metabolisms.

In this study, we observed that conidia of ΔFgPtc3-8 contained more lipid droplets than the wild-type progenitor. SAGE data also showed that many genes associated with lipid biosynthesis and metabolism were upregulated significantly in FgPtc3-8 (Table S2). To our knowledge, it is the first report that a PP2C protein is involved in lipid metabolism in fungi. In *Magnaporthe grisea*, lipid droplet is one of the major storage reserves in conidia [44]. During germination, lipid droplets are mobilized quickly from conidium to germ tube apex and incipient appressorium. Subsequently, lipid droplets are taken up by vacuole and degraded to generate glycerol in the appressorium [45]. It is commonly accepted that full use of storage reserves in conidium is important for a phytopathogenic fungus to establish initial infection on host plant. In *M. grisea*, a deletion mutant of fatty acid-oxidation enzyme gene showed a delay in lipid droplet mobilization and degradation. This blockage of lipid droplet degradation prevented *M. grisea* from infecting plant host [46]. In this study, we observed that ΔFgPtc3-8 accumulated numerous large lipid droplets in conidia, and these lipid droplets were degraded gradually during spore germination and hyphal growth. However, the mutant was not able to infect the host plant. Thus, we propose that different from *M. grisea*, the saprophytic fungus *F. graminearum* may not use the storage reserves of lipid droplets in conidia to establish its initial infection on wheat head.

In fungi, hyperosmotic stresses can activate the HOG pathway, which in turn increases the accumulation of glycerol to maintain the internal turgor pressure in fungal cells [36]. A genome-wide search for the HOG pathway-related genes in *F. graminearum* revealed that the fungus possesses many putative HOG elements, including one osmosensor histidine kinase FgOs-1 and 15 other histidine kinases, one histidine phosphotransfer protein FgHpt-1, two response regulators FgRrg-1 and FgRrg-2, and the downstream mitogen-activated protein kinase (MAPK) cascades (FgOs-4, FgOs-5 and FgOs-2) [47]. As expected, disruption of several genes (including *FgRRG1*, *FgOS4*, *FgOS5* and *FgOS2*) led to increased sensitivity of *F. graminearum* to osmotic stress [33], [47]. In contrast to these mutants, in current study, we found that ΔFgPtc3-8 showed increased tolerance to osmotic stress. This observation is in agreement with the finding that ΔFgPtc3-8 accumulated a higher level of glycerol than ΔFgOs2-4. Additionally, after treated with 1.2 M NaCl for 2 hours, expression of *FgPTC3* in the wild type strain was decreased by 74% (data not shown). These results led to our hypothesis that FgPtc3 may be a negative regulator of the HOG pathway. This inference was further supported by high levels of *FgOS2* expression in ΔFgPtc3-8. In *S. cerevisiae*, Ptc1 performs its functions on the HOG pathway through the adaptor protein Nbp2 [14]. In current study, using yeast two-hybrid analysis, we found that FgPtc3 does not physically interact with FgNbp2, FgOs5 and FgOs2. Furthermore, the deletion mutant of *FgNBP2* did not show recognizable changes in their morphological characters (Yun & Ma, unpublished data). These results indicate that FgNbp2 may not be an adaptor linking FgPtc3 to the HOG pathway in *F. graminearum*.

In addition to inactivating Hog1, the Ptc1 is also involved in the CWI pathway in *S. cerevisiae* [5]. This pathway is composed of several membrane sensors and the downstream MAPK cascades Pkc1, Bck1, Mkk1/2, and Slt2 [48]. Expression profile analysis showed that expressions of *SLT2* and several other CWI-related genes were induced in the *PTC1* mutant. Consistent with the gene expression data, higher levels of phosphorylated Slt2 protein were detected in the *PTC1* mutant [41]. To date, the CWI pathway in *F. graminearum* has not been well documented. A genome-wide search for the CWI pathway-related genes revealed that *F. graminearum* possesses several putative components of CWI pathway including FgMkk1 and FgSlt2. In current study, we observed that the *FgPTC3* mutant revealed increased resistance to cell wall damaging agents, caffeine and congo red. Furthermore, high expression levels of *FgMKK1*and *FgSLT2* genes were detected in the mutant. Since an antibody for detection of phosphorylated FgSlt2 presently unavailable, we were unable to analyze phosfphorylated profile of FgSlt2 in the ΔFgPtc3-8 mutant using the antibodies correspondingly yeast Slt2 protein. To gain insight into functions of FgPtc3, the *FgPTC3* gene was transferred into the yeast *PTC1* mutant, which rescued the partial growth defects of the mutant under cell wall damaging and other stress conditions. These results strongly indicate that FgPtc3 is involved in regulating the CWI pathway in *F. graminearum*.

## Materials and Methods

### Strains and culture conditions

*F. graminearum* wild-type strain PH-1 was used as a parental strain for transformation experiments. The wild-type strain and transformants generated in this study were grown on potato dextrose agar (PDA) (200 g potato, 20 g dextrose, 20 g agar, and 1 L water) or minimal medium (MM) (10 mM K_2_HPO_4_, 10 mM KH_2_PO_4_, 4 mM (NH_4_)_2_SO_4_, 2.5 mM NaCl, 2 mM MgSO_4_, 0.45 mM CaCl_2_, 9 µM FeSO_4_, 10 mM glucose, and 1 L water, pH 6.9) for mycelial growth tests, and in mung bean liquid (MBL) (40 g mung beans boiled in 1L water for 20 min, and filtered through cheesecloth) medium [49] for sporulation analysis.

### Sequence analysis of PP2C genes

The *FgPTC1* (FGSG_04111.3), *FgPTC3* (FGSG_10239.3), *FgPTC5* (FGSG_05205.3), *FgPTC5R* (FGSG_04026.3*)*, *FgPTC6* (FGSG_05162.3), *FgPTC7* (FGSG_05611.3) and *FgPTC7R* (FGSG_00435.3) were originally identified through homology searches of the *F. graminearum* genome sequence (available at http://www.broadinstitute.org/annotation/genome/fusarium_group/MultiHome.html) by using BLAST with the type 2C protein Ser/Thr phosphatases Ptc1 (SCRG_00513.1), Ptc2 (SCRG_04566.1), Ptc3 (SCRG_03018.1), Ptc4 (SCRG_02843.1), Ptc5 (SCRG_01488.1), Ptc6 (SCRG_05468.1) and Ptc7 (SCRG_04788.1) from *S. cerevisiae* as queries. To verify the existence and the size of introns in *FgPTC1*, *FgPTC3*, *FgPTC5*, *FgPTC5R*, *FgPTC6*, *FgPTC7* and *FgPTC7R*, RNA was extracted from mycelia of the wild-type strain PH-1 using the TaKaRa RNAiso Reagent (TaKaRa Biotech. Co., Dalian, China) and used for reverse transcription with a RevertAid H Minus First Strand cDNA Synthesis kit (Fermentas Life Sciences, Burlington, Canada) according to the manufacturer's instructions. Reverse transcription PCR of *FgPTC1*, *FgPTC3*, *FgPTC5*, *FgPTC5R*, *FgPTC6*, *FgPTC7*, and *FgPTC7R* cDNAs were performed using the primer pairs Fptc1-F1 + Fptc1-R1, Fptc3-F1 + Fptc3-R1, Fptc5a-F1 + Fptc5a-R1, Fptc5b-F1 + Fptc5b-R1, Fptc6-F1 + Fptc6-R1, Fptc7a-F1 + Fptc7a-R1 and Fptc7b-F1 + Fptc7b-R1 (Table S1), respectively. PCR amplifications were performed using the following parameters: initial denaturation at 95°C for 3 min, followed by 35 cycles of denaturation at 94°C for 40 s, annealing at 54°C for 40 s, extension at 72°C for 2 min, and final extension at 72°C for 10 min. The resultant PCR products were purified, cloned and sequenced.

### Construction of vectors for the deletion of PP2C genes

The *FgPTC1* gene disruption vector pCA-FgPtc1-Del was constructed by inserting two flanking sequences of *FgPTC1* gene into two sides of the *HPH* (hygromycin resistance) gene in the pBS-HPH1 vector [50]. The upstream flanking sequence fragment of *FgPTC1* was amplified from PH-1 genomic DNA using the primer pair P11+P12 (Table S1). The 981-bp fragment was inserted into *Xho*I-*Sal*I sites of the pBS-HPH1 vector to generate the plasmid pBS-FgPtc1-up. Subsequently, a 1013-bp downstream flanking sequence fragment of *FgPTC1* was amplified from the PH-1 genomic DNA using the primer pair P13+P14 (Table S1) and was inserted into the *Hind*III-*BamH*I site of pBS- FgPtc1-up vector to generate the plasmid pBS- FgPtc1-UD. Finally, the 3,497-bp fragment containing FgPtc1-upstream-HPH- FgPtc1-downstream cassette (Figure S1A) was obtained by digestion of plasmid pBS- FgPtc1-UD with *Xho*I and *BamH*I, and ligated into the *Xho*I-*BamH*I site in pCAMBIA 1300 (CAMBIA, Canberra, Australia). The resultant *FgPTC1* deletion vector pCA- FgPtc1-Del was transformed into *Agrobacterium tumefaciens* strain C_58_C_1_ by electroporation, the *A. tumefaciens*-mediated fungal transformation was performed as described previously [51]. Using the same strategy, vectors were constructed for the disruption of other six genes.

To construct double gene disruption vector pCA-FgPtc13-Del, a *Sal*I-*Hind*III *SUR* cassette was amplified from plasmid PCB1532 [52] with the primer pair sur-F + sur-R (Table S1), and cloned into the *Sal*I-*Hind*III site of pBS- FgPtc1-UD to create plasmid pBS- FgPtc1-UD-Sur. Finally, the 4,813-bp fragment containing FgPtc1-upstream-SUR- FgPtc1-downstream cassette was obtained by digestion of plasmid pBS- FgPtc1-UD-Sur with *Xho*I and *BamH*I, and ligated into the *Xho*I-*BamH*I site in pCAMBIA 1300. The resultant *FgPTC1* and *FgPTC3* double deletion vector pCA-FgPtc13-Del was transformed into *A. tumefaciens* strain C_58_C_1_ by electroporation. Transformation of ΔFgPtc3-8 with pCA-FgPtc13-Del was conducted as described below except that chlorimuron-ethy1 was used as a selection agent.

### Complementation of the *FgPTC3* gene deletion mutant

The *FgPTC3* deletion mutant (ΔFgPtc3-8) was complemented with a full-length *FgPTC3* gene, to confirm that the phenotype changes in *FgPTC3* deletion mutant were due to the disruption of the gene. The *FgPTC3* complement plasmid pCA-FgPtc3-C was constructed using the backbone of pCAMBIA1300. First, a *Xho*I-*Kpn*I *NEO* cassette containing a *trpC* promoter was amplified from plasmid pBS-RP-Red-A8-NEO [53] with primers neo-F + neo-R (Table S1), and cloned into the *Xho*I-*Kpn*I site of pCAMBIA1300 to create plasmid pCA-neo. Then, a full-length *FgPTC3* gene including 1,461-bp promoter region and 1082-bp terminator region was amplified from genomic DNA of the wild-type strain PH-1 using the primer pair P3-com-F + P3-com-R (Table S1), and subsequently cloned into the *Pst*I and *Hind*III sites of pCA-neo to generate the complement plasmid pCA-FgPtc3-C. Before plasmid pCA-FgPtc3-C was transformed into *A. tumefaciens* strain C_58_C_1_, the *FgPTC3* in this plasmid was sequenced to ensure flawlessness of the sequence. Transformation of ΔFgPtc3-8 with the full-length *FgPTC3* gene was conducted as described above except that geneticin was used as a selection agent.

### Mycelial growth and conidiation assays

Mycelial growth tests under different conditions were performed on PDA or MM plates supplemented with the following products: NaCl, KCl, LiCl, CaCl_2_, caffeine, congo red at concentrations indicated in figure legends. Each plate was inoculated with a 5-mm mycelial plug taken from the edge of a 3-day-old colony. Plates were incubated at 25°C for 4 d in the dark, and then colony diameter in each plate was measured and the original mycelial plug diameter (5 mm) subtracted from each measurement. The percentage of the mycelial radial growth inhibition (RGI) was calculated using the formula RGI = ((C–N)/(C–5))*100, where, C is colony diameter of the control, and N is that of a treatment. Each experiment was repeated three times.

For conidiation assays, ten mycelial plugs (5-mm in diameter) of each strain taken from the periphery of a 3-day-old colony were inoculated in a 50-ml flask containing 10 ml of MBL medium. The flasks were incubated at 25°C for 4 d in a shaker (180 rpm). For each strain, the number of conidia in the broth was determined using a hemacytometer. The experiment was repeated three times.

### Yeast strains and complementation assays

The yeast strain BY4741 (wild type) and a *PTC1* deletion mutant BY4741ΔPtc1 were ordered from EUROSCARF. The full-length *FgPTC3* cDNA was amplified using the primer pair YES2-ptc3-F + YES2-ptc3-R. The PCR product was digested with *Hind*III and *Sac*I, cloned into the pYES2 vector (Invitrogen), and transformed into the yeast mutant BY4741ΔPtc1. Yeast transformants were selected on synthetic medium lacking Ura (Clontech). Additionally, the wild type strain BY4741 and BY4741ΔPtc1 mutant transformed with empty pYES2 vector were used as controls. For the complementation assays, the yeast transformants were grown on SG medium (0.67% yeast nitrogen base without amino acids, 2% galactose, 1% raffinose, 2% agar) supplied with various stress agents including caffeine, calcofluor white (CFW), Congo red, CaCl_2_, and ZnCl_2_ at concentrations indicated in figure legends. The experiments were repeated three times.

### Microscopic examination of hyphal and conidial morphology

Hyphal and spore morphology of the PP2C gene mutants were examined with the Leica TCS SP5 imaging system. For transmission electron microscopy, spores were fixed with 2.5% glutaraldehyde for 24 h at 4°C, then samples were washed with 0.1 M PBS for three times, fixed in 1% osmic acid for 3 h at room temperature. After the fixed tissues were rinsed 3 times (15 min each) with 0.1 M PBS, and dehydrated in graded ethanol solutions, the samples were embedded in Lowicryl K4M resin (Electron Microscopy Sciences, Fort Washington, PA, USA). Ultrathin sections (70 nm) were cut from the embedded tissue blocks and mounted onto nickel grids before observation. The sections were examined under an electron microscope JEM-1200EX (JEOL, Japan).

### Histochemical analysis of lipid droplets

Lipid droplets in the conidia were visualized by staining with a Nile Red staining solution [44], [54] consisting of 20 mg/mL polyvinylpyrrolidone and 2.5 µg/mL Nile Red Oxazone (9-diethylamino-5H-benzo[α] phenoxazine-5-one, Sigma) in 50 mM Tris-maleate buffer (pH 7.5). Briefly, after incubation in MBL medium for 3 d, conidia of each strain were harvested and mounted in the Nile Red staining solution. Within a few seconds, lipid droplets began to fluoresce when viewed under a microscope with episcopic fluorescence attachment.

### Determination of intracellular glycerol accumulation

Each strain was grown in potato dextrose broth (PDB) for 2 days at 25°C in a shaker. After treated with 1.2 M NaCl for 2 h, mycelia of each strain were harvested and ground in liquid nitrogen. Then, mycelial powder (100 mg) was transferred to a 2-ml microcentrifuge tube containing 0.1 ml glycerol extraction buffer (Shanghai Chaoyan Biotechnology Co.). After vortexed three times for 30 s each, the tubes were centrifuged at 5000 *g* for 20 min. The resulting supernatant was transferred to a new tube, and 10 µl of each supernatant was mixed with 190 µl detection buffer of a glycerol assay kit (Shanghai Chaoyan Biotechnology Co.). After the mixture was incubated at 37°C for 15 min, the glycerol concentration was determined by a spectrophotometer (SPECTRAmax Plus) at 550 nm. The experiment was repeated twice.

### Yeast two hybrid analysis

To construct plasmids for yeast two hybrid screen analysis, the coding sequence of the full length *FgOS2, FgPTC3*, *FgNBP2*, or *FgOS5* was amplified from the cDNA of PH-1. The *FgOS2* and *FgNBP2* fragments were inserted into the *Nde*I-*BamH*I sites of the yeast GAL4 binding domain vector pGBKT7 and GAL4 activation domain vector pGADT7 (Clontech, Mountain View, CA, USA), respectively. The *FgPTC3* and *FgOS5* PCR fragments were inserted into the *Nde*I-*Sma*I sites of the yeast GAL4 binding domain vector pGBKT7 and GAL4 activation domain vector pGADT7, respectively. The yeast two hybrid plasmids AD-FgOs2 +BD-FgPtc3, AD-FgOs5 +BD-FgPtc3, AD-FgNbp2 +BD-FgPtc3 were co-transformed into the *S. cerevisiae* reporter strain AH109 according to LiAc/SS-DNA/PEG transformation procedure [55]. The pair of plasmid pGBKT7-53 and pGADT7 was served as a positive control. The pairs of plasmids pGBKT7-Lam and pGADT7, pGBKT7 and pGADT7-FgOs2, pGBKT7 and pGADT7-FgNbp2, pGADT7 and pGBKT7-FgPtc3 were used as negative controls. Transformants were grown at 30°C for 72 h on synthetic medium lacking Leu and Trp, and then transferred to the medium lacking His, Leu and Trp and containing 5 mM 3-aminotriazole (3-AT) to identify binding activity. Three independent experiments were performed to confirm yeast two hybrid results.

### SAGE analysis of gene expression profiling in ΔFgPtc3-8

The wild type progenitor and ΔFgPtc3-8 were grown in PDB for 2 days at 25°C in a shaker. Then mycelia of each strain were harvested and used for RNA extraction. The library constructions used for SAGE analysis were performed from the total RNA of wild-type strain and ΔFgPtc3-8 mutant using the kit for preparing samples for digital gene expression-Tag profiling with DpnII (Illumina Inc., California, USA) according to the manufacturer's protocol. The experiment was performed by (BGI Co., Shenzhen, China) using Illumina Cluster Station and Illumina HiSeq (TM) 2000 System. Since tags detected by SAGE with a frequency less than 3 transcripts per million (tpm) may not be reliable [56], only tags with a frequency ≥3 tpm were used in data analysis in this study. The unique tags were then aligned to all the known transcripts of *F. graminearum* using Novoalign aligner (Novocraft Technologies, Kuala Lumpur, Malaysia). The frequencies of each SAGE tag in the *FgPTC3* deletion mutant ΔFgPtc3-8 and the wild-type strain PH-1 were compared, and the statistical significance (*P* value) was calculated according to the Audic and Claverie test using the program IDEG6 [57]. The *P* value is a measure of the confidence that the gene is differentially expressed in the two compared samples.

### Pathogenicity assays on flowering wheat heads

After incubation in MBL medium for 4 days, conidia of each strain were collected by filtration through three layers of gauze and subsequently resuspended in sterile distilled water to a concentration of 1×10^5^ conidia/ml. A10-µl aliquot of conidial suspension was injected into a floret in the central section spikelet of single flowering wheat heads of susceptible cultivar Zimai12. There were ten replicates for each strain. After inoculation, the plants were kept at 22±2°C under 95–100% humidity. Fifteen days after inoculation, the infected spikelets in each inoculated wheat head were recorded. The experiment was repeated for four times.

### Analysis of DON production and expression level of *TRI5*

A 30-g aliquot of healthy wheat kernels was sterilized and inoculated with 1 ml spore suspension (10^6^ spores/ml) of the wild-type strain PH-1, the complemented strain ΔFgPtc3-8C, ΔFgPtc3-8, ΔFgPtc13-6, and other six genes deletion mutants. After incubation at 25°C for 20 days, DON was extracted using a previously described protocol [58], and the amount of *F. graminearum* DNA in each sample was determined using a quantitative real-time PCR method [59]. The DON extracts were purified with PuriTox^SR^ DON column TC-T200 (Trilogy analytical laboratory), and the amount of DON (per mg fungal DNA) in each sample was determined by using a HPLC system Waters 1525. The experiment was repeated three times, and data were analyzed using analysis of variance (SAS version 8.0; SAS Institute, Cary, NC).

To determine expression level of *TIR5*, the mycelia of the wild-type progenitor PH-1, and the ΔFgPtc3-8 were inoculated into GYEP medium (5% glucose, 0.1% yeast extract, 0.1% peptone) and cultured for 2 days at 25°C in the dark. Total RNA was extracted from mycelia of each sample, the expression of *TRI5* was determined using a quantitative real-time PCR method. The experiment was repeated three times.

## Supporting Information

Figure S1 **Schematic representation of the** ***FgPTC1*** **disruption strategy.** (**A**) *FgPTC1* and hygromycin resistance cassette [*HPH*] are denoted by large black and gray arrows, respectively. Annealing sites of primers P11, P12, P13, P14, P15 and P16 are indicated with arrows (see Table S1 for the primer sequences). (**B**) A 981-bp upstream fragment of *FgPTC1* was used as a probe in Southern blot hybridization analysis. Genomic DNA preparations of the wild-type PH-1, the *FgPTC1* deletion mutant ΔFgPtc1-5, and the *FgPTC1* and *FgPTC3* double mutant ΔFgPtc13-6 were digested with *Nco*I.
(TIF)

Figure S2 **Phylogenetic analysis and alignments of seven type 2C Ser/Thr phosphatases from** ***F. graminearum and S. cerevisiae*** **.** (**A**) Phylogenetic analysis of amino acid sequence of seven type 2C Ser/Thr phosphatases from *F. graminearum* and *S. cerevisiae*. (**B**) Alignments of amino acid sequences of seven PP2C in *F. graminearum* with those of *S. cerevisiae*.
(TIF)

Figure S3 **Schematic representation of the** ***FgPTC3*** **disruption strategy.** (**A**) *FgPTC3* and hygromycin resistance cassette [*HPH*] are denoted by large black and gray arrows, respectively. Annealing sites of PCR primers are indicated with arrows [see Table S1 for the primer sequences]. (**B**) A 977-bp downstream fragment of *FgPTC3* was used as a probe in Southern blot hybridization analysis. Genomic DNA preparations of the wild-type PH-1, the *FgPTC3* deletion mutant ΔFgPtc3-8, and the complement strain ΔFgPtc3-8C were digested with *Pvu*I.
(TIF)

Figure S4 **Virulence of the wild-type strain PH-1 and other six PP2C mutants on wheat heads.** Wheat heads were point-inoculated with conidial suspension of each strain, and infected wheat heads were examined 15 days after inoculation.
(TIF)

Figure S5 **Yeast two-hybrid analysis of the interaction between FgPtc3 and FgOs2, FgNbp2.** The pair of plasmids pGBKT7-53 and pGADT7 was served as a positive control. The pairs of plasmids pGBKT7-Lam and pGADT7, pGBKT7 and pGADT7-FgOs2, pGBKT7 and pGADT7-FgNbp2, pGADT7 and pGBKT7-FgPtc3 were used as negative controls. Growth of the transformed yeast was assayed on the medium containing 5 mM 3-aminotriazole [3-AT], but lacking His, Leu and Trp. Columns in each panel represent serial decimal dilution.
(TIF)

Figure S6 **The genes involved in peroxisome biogenesis (A), mitogen-activated protein kinase (MAPK) signaling pathway (B) and target of rapamycin (TOR) signaling pathway (C) were up- or down-regulated in the** ***FgPTC3*** **mutant.** The up- and down-regulated genes are indicated in red- and green- boxes, respectively.
(TIF)

Table S1 **Oligonucleotide primers used in this study.**
(DOC)

Table S2 **Expression changes of the genes involved in fatty acid biosynthesis and metabolism in** ***F. graminearum FgPTC3*** **deletion mutant ΔFgPtc3-8 detected by serial analysis of gene expression method.**
(DOC)
