# Supplementary figures and images for: A Type 2C Protein Phosphatase FgPtc3 Is Involved in Cell Wall Integrity, Lipid Metabolism, and Virulence in Fusarium graminearum
Source: PLoS One. 2011 Sep 28;6(9):e25311. doi: 10.1371/journal.pone.0025311 (PMC3182220; doi:10.1371/journal.pone.0025311)

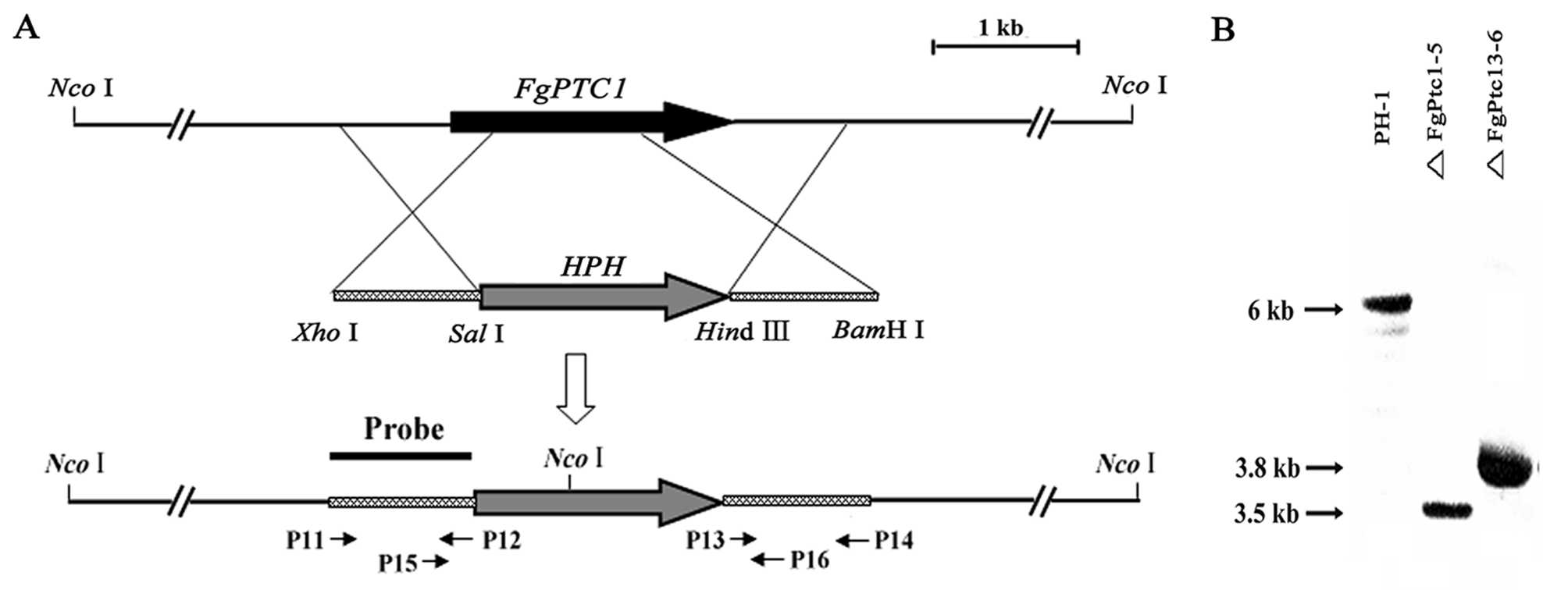

Supplement: Figure S1 — Schematic representation of the FgPTC1 disruption strategy. (A) FgPTC1 and hygromycin resistance cassette [HPH] are denoted by large black and gray arrows, respectively. Annealing sites of primers P11, P12, P13, P14, P15 and P16 are indicated with arrows (see Table S1 for the primer sequences). (B) A 981-bp upstream fragment of FgPTC1 was used as a probe in Southern blot hybridization analysis. Genomic DNA preparations of the wild-type PH-1, the FgPTC1 deletion mutant ΔFgPtc1-5, and the FgPTC1 and FgPTC3 double mutant ΔFgPtc13-6 were digested with NcoI. (TIF) [file pone.0025311.s001.tif]

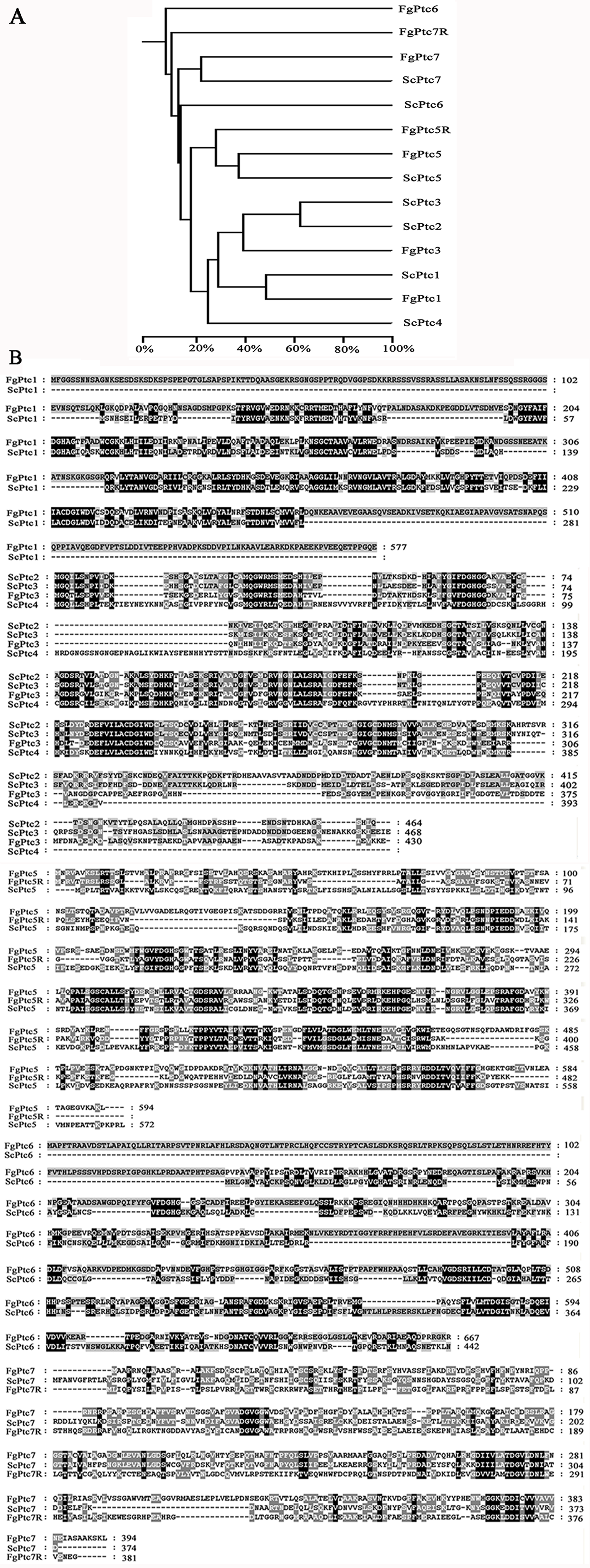

Supplement: Figure S2 — Phylogenetic analysis and alignments of seven type 2C Ser/Thr phosphatases from F. graminearum and S. cerevisiae . (A) Phylogenetic analysis of amino acid sequence of seven type 2C Ser/Thr phosphatases from F. graminearum and S. cerevisiae. (B) Alignments of amino acid sequences of seven PP2C in F. graminearum with those of S. cerevisiae. (TIF) [file pone.0025311.s002.tif]

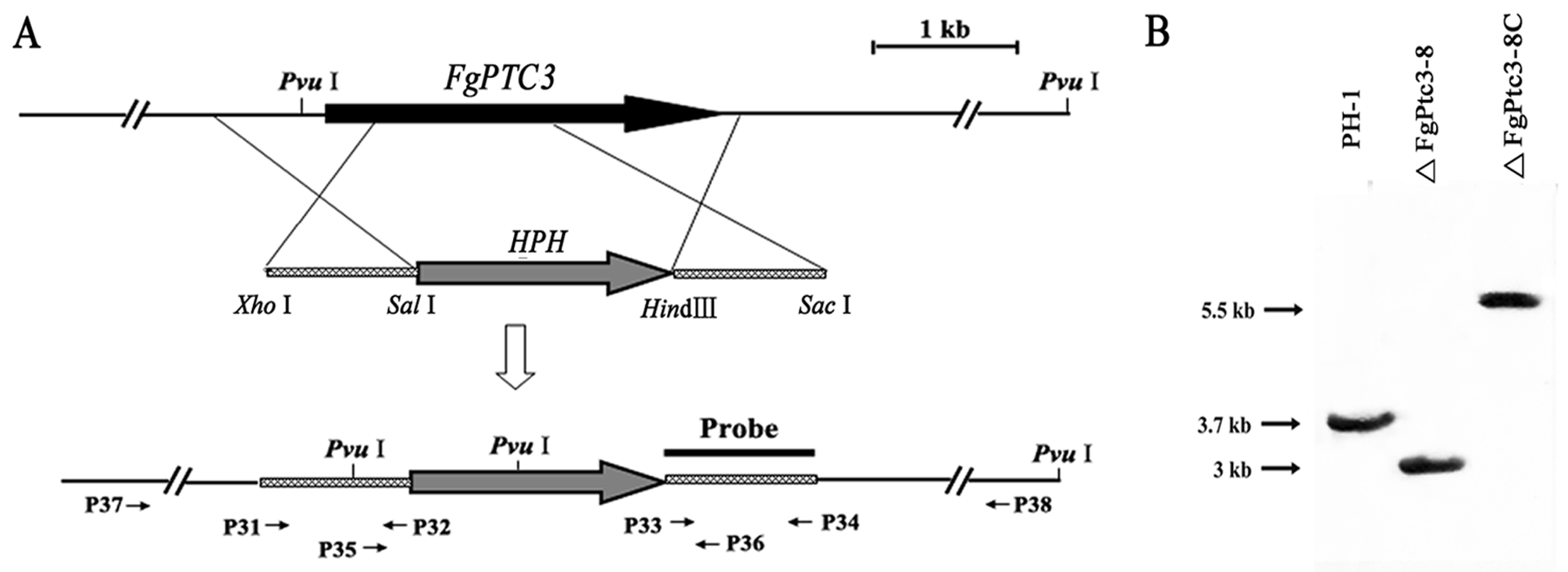

Supplement: Figure S3 — Schematic representation of the FgPTC3 disruption strategy. (A) FgPTC3 and hygromycin resistance cassette [HPH] are denoted by large black and gray arrows, respectively. Annealing sites of PCR primers are indicated with arrows [see Table S1 for the primer sequences]. (B) A 977-bp downstream fragment of FgPTC3 was used as a probe in Southern blot hybridization analysis. Genomic DNA preparations of the wild-type PH-1, the FgPTC3 deletion mutant ΔFgPtc3-8, and the complement strain ΔFgPtc3-8C were digested with PvuI. (TIF) [file pone.0025311.s003.tif]

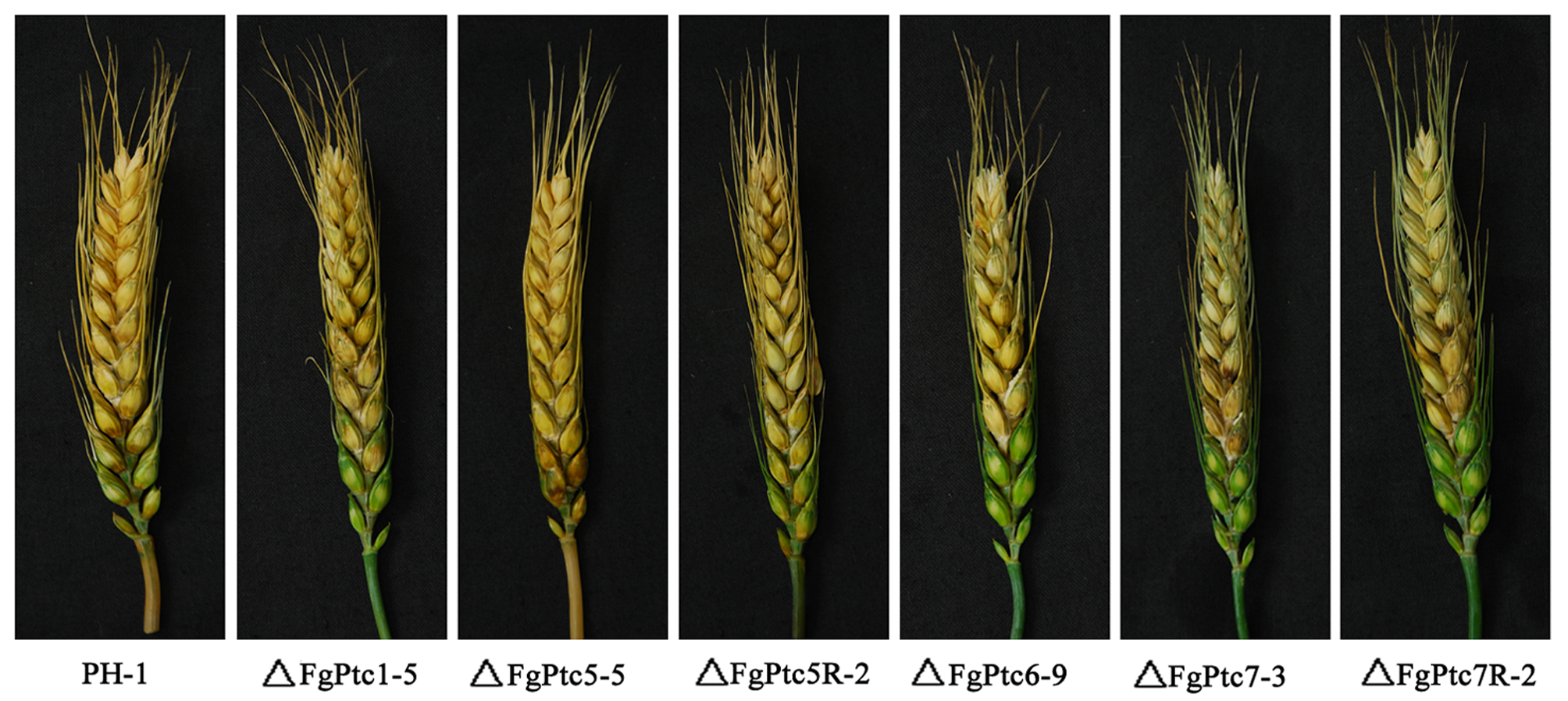

Supplement: Figure S4 — Virulence of the wild-type strain PH-1 and other six PP2C mutants on wheat heads. Wheat heads were point-inoculated with conidial suspension of each strain, and infected wheat heads were examined 15 days after inoculation. (TIF) [file pone.0025311.s004.tif]

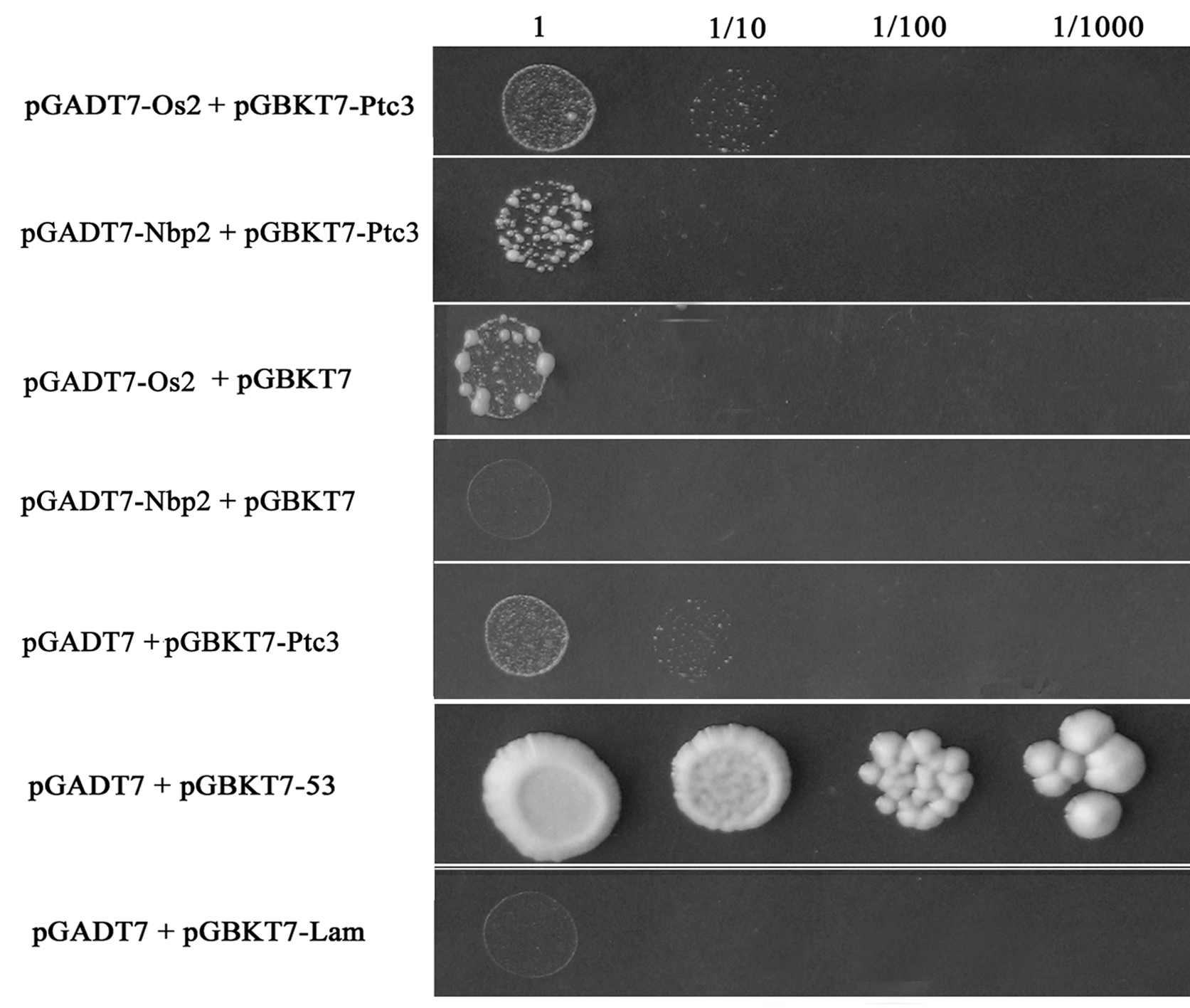

Supplement: Figure S5 — Yeast two-hybrid analysis of the interaction between FgPtc3 and FgOs2, FgNbp2. The pair of plasmids pGBKT7-53 and pGADT7 was served as a positive control. The pairs of plasmids pGBKT7-Lam and pGADT7, pGBKT7 and pGADT7-FgOs2, pGBKT7 and pGADT7-FgNbp2, pGADT7 and pGBKT7-FgPtc3 were used as negative controls. Growth of the transformed yeast was assayed on the medium containing 5 mM 3-aminotriazole [3-AT], but lacking His, Leu and Trp. Columns in each panel represent serial decimal dilution. (TIF) [file pone.0025311.s005.tif]

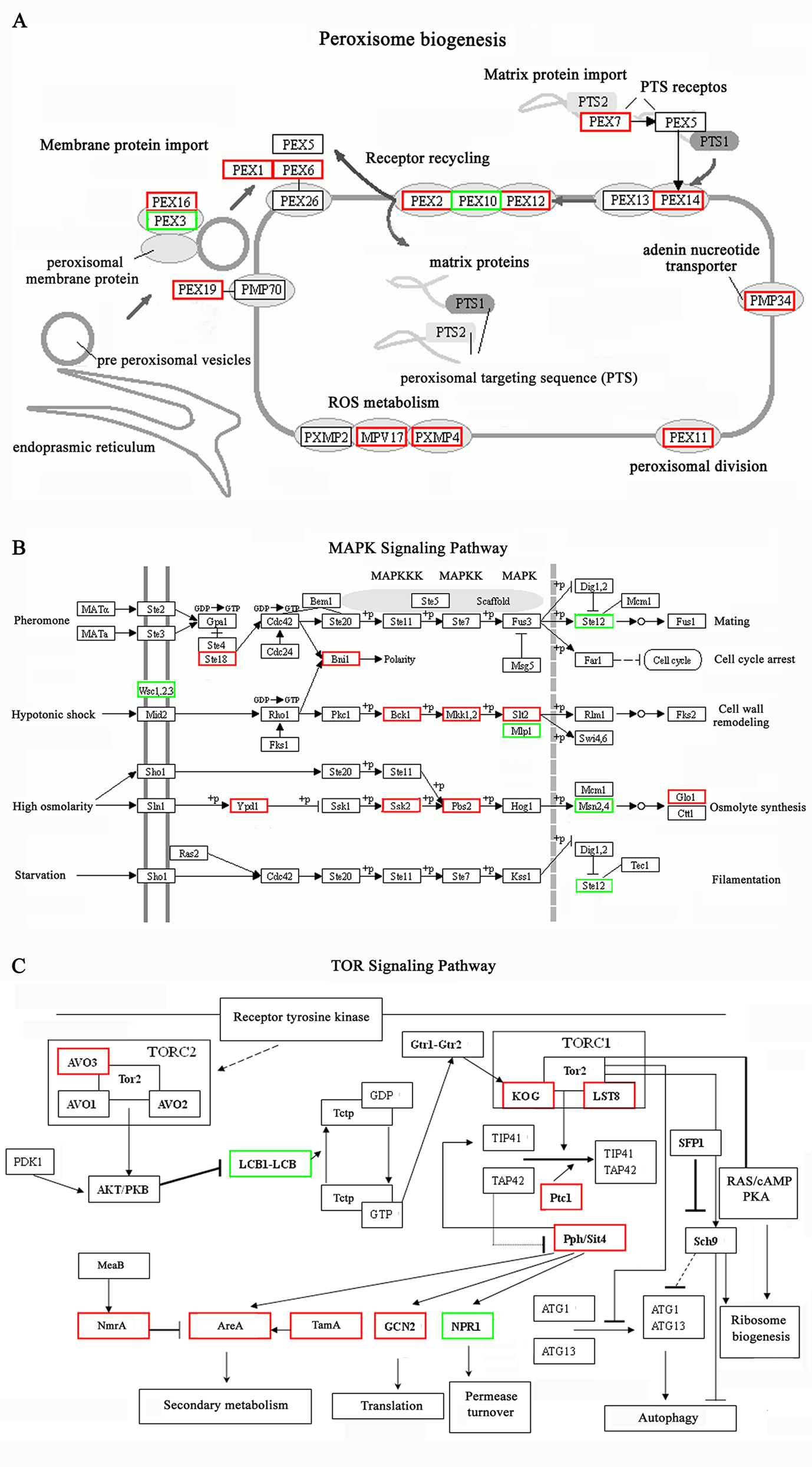

Supplement: Figure S6 — The genes involved in peroxisome biogenesis (A), mitogen-activated protein kinase (MAPK) signaling pathway (B) and target of rapamycin (TOR) signaling pathway (C) were up- or down-regulated in the FgPTC3 mutant. The up- and down-regulated genes are indicated in red- and green- boxes, respectively. (TIF) [file pone.0025311.s006.tif]
