# Supplementary material for: A Type 2C Protein Phosphatase FgPtc3 Is Involved in Cell Wall Integrity, Lipid Metabolism, and Virulence in Fusarium graminearum
Source: PLoS One. 2011 Sep 28;6(9):e25311. doi: 10.1371/journal.pone.0025311 (PMC3182220; doi:10.1371/journal.pone.0025311)
Supplement: Table S1 — Oligonucleotide primers used in this study. (DOC) [file pone.0025311.s007.doc]

Table S1. Oligonucleotide primers used in this study

| Primer code | Primer | Sequence (5’-3’)^a^ | Relevant characteristics |
| --- | --- | --- | --- |
| 1 | Fptc1-F1 | catatgATGTTTGGCGGCAGTTCCA | A pair of PCR primers for amplification of the full cDNA sequence of the *FgPTC1* gene |
| 2 | Fptc1-R1 | cccgggTTATTCCTGCCCAGGAGGTGT |
|  |  |  |  |
| 3 | P11 | ATctcgagTCATTCCCTTGTGGCGTCTA | A pair of PCR primers for amplification of the 981-bp *FgPTC1* upstream fragment for construction of the gene deletion vector |
| 4 | P12 | ATgtcgacTTTCTGAAGAGATGTCTGCGA |
|  |  |  |  |
| 5 | P13 | ATaagcttTATGCGCTGAATCGCTTCA | A pair of PCR primers for amplification of the 1013-bp *FgPTC1* downstream fragment for construction of the gene deletion vector |
| 6 | P14 | ATggatccAATCAACGTTATGGGGCCTA |
|  |  |  |  |
| 7 | P15 | ACCAACTCGCCAGGATGTT | A pair of PCR primers for identification of *FgPTC1* deletion mutants |
| 8 | P16 | GGGTGCTATACCTTCTGCAAT |
|  |  |  |  |
| 9 | P1-probe-F | ATctcgagTCATTCCCTTGTGGCGTCTA | PCR primers to amplify the 981-bp *FgPTC1* fragment used as the probe for Southern blot analysis |
| 10 | P1-probe-R | ATgtcgacTTTCTGAAGAGATGTCTGCGA |
|  |  |  |  |
| 11 | Fptc3-F1 | catatgATGGGTCAGACACTGTCAGAG | PCR primers for amplification of full cDNA sequence of the *FgPTC3* gene |
| 12 | Fptc3-R1 | cccgggCTACTCCTTCTTCACTTCGGTCT |
|  |  |  |  |
| 13 | P31 | ATctcgagACCTCCAGTGCTCAAAGGCTT | PCR primers to amplify the 1035-bp *FgPTC3* upstream fragment for construction of the gene deletion vector |
| 14 | P32 | ATgtcgacTGCATAGCCGATACACCGTA |
|  |  |  |  |
| 15 | P33 | ATaagcttAGAGCAGCATGATCTCACAGA | PCR primers to amplify the 977-bp *FgPTC3*  downstream fragment for construction of the deletion vector |
| 16 | P34 | ATgagctcCGATCCAGATTTGACACCAA |
|  |  |  |  |
| 17 | P35 | TCAGACACTGTCAGAGCCCGT | PCR primers for identification of *FgPTC3* deletion transformants |
| 18 | P36 | GCTTGGGAGGATTGGCAAT |
|  |  |  |  |
| 19 | P3-com-F | ATctgcagCTAGGACATGCAGAAGCATCA | A pair of PCR primers to amplify the full *FgPTC3* including 1,461-bp up- and 1,082-bp down-fragments |
| 20 | P3-com-R | ATaagcttATGAGCGTGAAGGCGATGTA |
|  |  |  |  |
| 21 | Yes2-ptc3-F | aagcttATGGGTCAGACACTGTCAGAG | PCR primers for amplification of full cDNA sequence of the *FgPTC3* gene |
| 22 | Yes2-ptc3-R | gagctcCTACTCCTTCTTCACTTCGGTCT |
|  |  |  |  |
| 23 | Fptc5a-F1 | ATGAATCGAGTTGCCGTCAAATC | A pair of PCR primers for amplification of the full cDNA sequence of the *FgPTC5* gene |
| 24 | Fptc5a-R1 | TTACAGCTTCGCCTTGACGCCC |
|  |  |  |  |
| 25 | P51 | ATctcgagAAGAAGTGAACCTCGGCTGAA | A pair of PCR primers for amplification of the 564-bp *FgPTC5* upstream fragment for construction of the gene deletion vector |
| 26 | P52 | ATgtcgacCAGGGGAGCTTGGAGCTAA |
|  |  |  |  |
| 27 | P53 | ATggatccTTCTTGGTGGATTGGAGCCTA | A pair of PCR primers for amplification of the 513-bp *FgPTC5* downstream fragment for construction of the gene deletion vector |
| 28 | P54 | ATgagctcACACCTGCTCGTCATTGTTG |
|  |  |  |  |
| 29 | P55 | GCCTGGTACTCCTACAACAGC | A pair of PCR primers for identification of *FgPTC5* deletion mutants |
| 30 | P56 | TTCACGGGCGACATAGTTGAT |
|  |  |  |  |
| 31 | Fptc5b-F1 | ATGTTTCGCGTAACCACCAGGA | A pair of PCR primers for amplification of the full cDNA sequence of the *FgPTC5R* gene |
| 32 | Fptc5b-R1 | TCACTTCTTCTCATAAGGATCC |
|  |  |  |  |
| 33 | P5R1 | ATctcgagAGCAGCGCCGACAAGAAAA | A pair of PCR primers for amplification of the 813-bp *FgPTC5R* upstream fragment for construction of the gene deletion vector |
| 34 | P5R2 | ATgtcgacGCACCCTTCACTTGATGATTT |
|  |  |  |  |
| 35 | P5R3 | ATctgcagATAAAGCATGGCAGAGACGA | A pair of PCR primers for amplification of the 976-bp *FgPTC5R* downstream fragment for construction of the gene deletion vector |
| 36 | P5R4 | ATggatccTCGTTGGATGCCATGTGTTT |
|  |  |  |  |
| 37 | P5R5 | TTCCGGGGCTTACATCTTC | A pair of PCR primers for identification of *FgPTC5R* deletion mutants |
| 38 | P5R6 | TGCCGTAGTAATCGTCCTGGA |
|  |  |  |  |
| 39 | Fptc6-F1 | ATGGCTCCGTTTACTCGCGCC | A pair of PCR primers for amplification of the full cDNA sequence of the *FgPTC6* gene |
| 40 | Fptc6-R1 | TCATCTCTTCCCTCGTCGTG |
|  |  |  |  |
| 41 | P61 | ATctcgagATAGGTTGCAGGTGGGATGA | A pair of PCR primers for amplification of the 614-bp *FgPTC6* upstream fragment for construction of the gene deletion vector |
| 42 | P62 | ATgtcgacTGTTTGTATGGGTTTGTTCGC |
|  |  |  |  |
| 43 | P63 | ATggatccTCATGTTGGAGACAGCCGTAT | A pair of PCR primers for amplification of the 572-bp *FgPTC6* downstream fragment for construction of the gene deletion vector |
| 44 | P64 | ATtctagaTCGTCATAATCACTCAAGCCA |
|  |  |  |  |
| 45 | P65 | CGAGCTTGTCCGACAAATC | A pair of PCR primers for identification of *FgPTC6* deletion mutants |
| 46 | P66 | TGTGTATTCGCTCCCCATGTA |
|  |  |  |  |
| 47 | Fptc7a-F1 | ATGGCCGCACCTCGCAACCAAC | A pair of PCR primers for amplification of the full cDNA sequence of the *FgPTC7* gene |
| 48 | Fptc7a-R1 | TCATAGCTTGCTCTTTGCAGCAG |
|  |  |  |  |
| 49 | P71 | ATctcgagTTCTGGCCGTTGATGTTCAT | A pair of PCR primers for amplification of the 929-bp *FgPTC7* upstream fragment for construction of the gene deletion vector |
| 50 | P72 | ATgtcgacATCGCGAACGGAGAAACAAA |
|  |  |  |  |
| 51 | P73 | ATctgcagTAACTCTGGACTCGTGACCAA | A pair of PCR primers for amplification of the 938-bp *FgPTC7* downstream fragment for construction of the gene deletion vector |
| 52 | P74 | ATggatccTTCCAGCGCTTAGGCCTGTTA |
|  |  |  |  |
| 53 | P75 | TCGTGGTTCTAATTGCTTCAG | A pair of PCR primers for identification of *FgPTC7* deletion mutants |
| 54 | P76 | TGAACTTCCTTGGCAAATGG |
|  |  |  |  |
| 55 | Fptc7b-F1 | ATGCTCATTCAACCATATTCG | A pair of PCR primers for amplification of the full cDNA sequence of the *FgPTC7R* gene |
| 56 | Fptc7b-R1 | TTAACCCTCATTCTCCACACAT |
|  |  |  |  |
| 57 | P7R1 | ATctcgagGCAACCTTCACACTGGAGACA | A pair of PCR primers for amplification of the 984-bp *FgPTC7R* upstream fragment for construction of the gene deletion vector |
| 58 | P7R2 | ATgtcgacTACAAGCCTGATGCTGATGCT |
|  |  |  |  |
| 59 | P7R3 | ATctgcag GGATCTCATTGAAGCAGCCAA | A pair of PCR primers for amplification of the 939-bp *FgPTC7R* downstream fragment for construction of the gene deletion vector |
| 60 | P7R4 | ATtctagaCTTTTCCCACCAAATTGTCG |
|  |  |  |  |
| 61 | P7R5 | ATTTATTCAATGTGGCTGCTG | A pair of PCR primers for identification of *FgPTC7R* deletion mutants |
| 62 | P7R6 | TTTGCGACAACTTACCCAAGA |
|  |  |  |  |
| 63 | Fos2-F1 | catatgATGGCCGAGTTTGTACGC | A pair of PCR primers for amplification of the full cDNA sequence of the *FgOS2* gene |
| 64 | Fos2-R1 | ggatccCTATTGTCCATTAAACTGCTCTTC |
|  |  |  |  |
| 65 | O21 | ATggatccAACCAAAATGTCGCCGTCA | PCR primers to amplify the 535-bp *FgOS2* upstream fragment for construction of the gene deletion vector |
| 66 | O22 | ATaagcttTGAGTCCACGCTGTAGACGAA |
|  |  |  |  |
| 67 | O23 | ATgtcgacTGAGCATGCACATGCAAGAA | PCR primers to amplify the 646-bp *FgOS2* downstream fragment for construction of the deletion vector |
| 68 | O24 | ATctcgagTCACAAATATGTGCGTATGCA |
|  |  |  |  |
| 69 | O25 | ATTATTGACTTCGCGCCCT | PCR primers for identification of *FgOS2* deletion transformants |
| 70 | O26 | TAAAAAAGCTCCTCTTGCCC |
|  |  |  |  |
| 71 | Os2-F | TTGTCAAGTCGCTACCCAAG | PCR primers for amplification of the partial *FgOS2* gene in quantitative real-time PCR assays |
| 72 | Os2-R | ATGTATCAACAGGCAGATCGG |
|  |  |  |  |
| 73 | Fnbp-F1 | catatgATGTTCCCCTCGACTCTCTCG | A pair of PCR primers for amplification of the full cDNA sequence of the *FgNBP2* gene |
| 74 | Fnbp-R1 | ggatccTATATTGAATCATGAGCGTCTC |
|  |  |  |  |
| 75 | Fos5-F1 | catatgATGTCCGCCTCCGACTCGGTTCCG | A pair of PCR primers for amplification of the full cDNA sequence of the *FgOS5* gene |
| 76 | Fos5-R1 | cccgggCTAGGCGTCATTTGGGCTTGAGATA |
|  |  |  |  |
| 77 | Tri5-F | TCACCCAGGAAACCCTACACT | PCR primers for amplification of the partial *TRI5* gene in quantitative real-time PCR assays |
| 78 | Tri5-R | ACGTTTGCCAGTTGTGCAA |
|  |  |  |  |
| 79 | Mkk1-F | GGATTCAACAAAGAGTGCGCT | PCR primers for amplification of the partial *FgMKK1* gene in quantitative real-time PCR assays |
| 80 | Mkk1-R | ACCGAAATCGCAAAGCTTGA |
|  |  |  |  |
| 81 | Slt2-F | TTCTTCACATTCTCGGAACCC | PCR primers for amplification of the partial *FgSLT2* gene in quantitative real-time PCR assays |
| 82 | Slt2-R | CATCCAAAATCATACCACGCA |
|  |  |  |  |
| 83 | Sur-F | ACGTGCCAACGCCACAGT | PCR primers for amplification of chlorimuron-ethyl resistance gene (*sur*) |
| 84 | Sur-R | ACGTGAGAGCATGCAATTCC |
|  |  |  |  |
| 85 | Hph-F | GACGTTGTAAAACGACGGCC | PCR primers for amplification of hygromycin resistance gene (*hph*) |
| 86 | Hph-R | AATTCGTCGACGTTAACTGGCTG |
|  |  |  |  |
| 87 | Neo-F | ATctcgagGGAGGTCAACACATCAATGCT | PCR primers for amplification of neomycin resistance gene (*neo*) |
| 88 | Neo-R | ATggtaccTCAGAAGAACTCGTCAAGAAG |
|  |  |  |  |
| 89 | actin-F | ATCCACGTCACCACTTTCAA | PCR primers for amplification of the reference actin gene in quantitative real-time PCR assays |
| 90 | actin-R: | TGCTTGGAGATCCACATTTG |
|  |  |  |  |

^a^ The respective restriction enzyme sites included in primers are listed in lowercase in the sequence.
