# Supplementary material for: A Type 2C Protein Phosphatase FgPtc3 Is Involved in Cell Wall Integrity, Lipid Metabolism, and Virulence in Fusarium graminearum
Source: PLoS One. 2011 Sep 28;6(9):e25311. doi: 10.1371/journal.pone.0025311 (PMC3182220; doi:10.1371/journal.pone.0025311)
Supplement: Table S2 — Expression changes of the genes involved in fatty acid biosynthesis and metabolism in F. graminearum FgPTC3 deletion mutant ΔFgPtc3-8 detected by serial analysis of gene expression method. (DOC) [file pone.0025311.s008.doc]

**Table S2. Expression changes of the genes involved in fatty acid biosynthesis and metabolism in *F. graminearum* *FgPTC3* deletion mutant ΔFgPtc3-8 detected by serial analysis of gene expression method.**

| Pathway | Accession number | Putative function | Fold change in gene expression^a^ |
| --- | --- | --- | --- |
| Fatty acid biosynthesis | FGSG_05322 | fatty acid synthase subunit beta dehydratase | 0.18 |
|  | FGSG_05321 | fatty acid synthase subunit alpha reductase | 0.15 |
|  | FGSG_07226 | 3-oxoacyl-[acyl-carrier-protein] synthase, mitochondrial precursor | 2.37 |
|  | FGSG_02324 | hypothetical protein similar to type I polyketide synthase | 2.33 |
|  | FGSG_02210 | conserved hypothetical protein | 17.11 |
|  | FGSG_07223 | hypothetical protein similar to short chain dehydrogenase family protein | 9.61 |
|  | FGSG_03838 | conserved hypothetical protein | 14.37 |
|  | FGSG_03375 | conserved hypothetical protein (953 nt);Pfam:PF00106.17 | 14.63 |
|  | FGSG_11409 | conserved hypothetical protein | 13.43 |
|  | FGSG_01857 | hypothetical protein similar to 3-oxoacyl-acyl-carrier-protein reductase | 8.91 |
|  | FGSG_10026 | hypothetical protein similar to 3-oxoacyl-(acyl-carrier-protein) reductase | 3.98 |
|  | FGSG_08816 | conserved hypothetical protein | 7.96 |
| Fatty acid metabolism | FGSG_07833 | conserved hypothetical protein | 10.39 |
|  | FGSG_01419 | hypothetical protein similar to AMP-binding protein | 20.07 |
|  | FGSG_07277 | conserved hypothetical protein | 14.76 |
|  | FGSG_08843 | hypothetical protein similar to AMP dependent CoA ligase | 14.68 |
|  | FGSG_13860 | conserved hypothetical protein | 8.93 |
|  | FGSG_09424 | hypothetical protein similar to fadD35 | 9.04 |
|  | FGSG_07659 | hypothetical protein similar to fadD36 | 5.98 |
|  | FGSG_01415 | conserved hypothetical protein | 3.28 |
|  | FGSG_08543 | hypothetical protein similar to Fum16p | 3.34 |
|  | FGSG_03363 | hypothetical protein similar to Fum16p | 0.17 |
|  | FGSG_02287 | hypothetical protein similar to acyl-CoA oxidase | 10.95 |
|  | FGSG_02379 | conserved hypothetical protein | 14.21 |
|  | FGSG_12573 | hypothetical protein similar to enoyl-CoA hydratase/isomerase family protein | 11.89 |
|  | FGSG_05551 | hypothetical protein similar to peroxisomal D3,D2-enoyl-CoA isomerase | 20.66 |
|  | FGSG_09979 | hypothetical protein similar to enoyl-CoA hydratase/isomerase family protein | 17.35 |
|  | FGSG_12529 | predicted protein | 4.08 |
|  | FGSG_13880 | conserved hypothetical protein | 6.13 |
|  | FGSG_13111 | enoyl-CoA hydratase, mitochondrial precursor | 6.45 |
|  | FGSG_03244 | hypothetical protein similar to enoyl-CoA hydratase/isomerase family protein | 6.19 |
|  | FGSG_07019 | hypothetical protein similar to mitochondrial 3-hydroxyisobutyryl-CoA hydrolase | 6.5 |
|  | FGSG_03546 | hypothetical protein similar to dehydrogenase | 4.59 |
|  | FGSG_00809 | 3-hydroxyacyl-CoA dehydrogenase type-2 | 10.92 |
|  | FGSG_09503 | 3-ketoacyl-CoA thiolase | 15.6 |
|  | FGSG_04243 | 3-ketoacyl-CoA thiolase | 16.11 |
|  | FGSG_13398 | 3-ketoacyl-CoA thiolase B | 5.07 |
|  | FGSG_01581 | conserved hypothetical protein | 6.26 |
|  | FGSG_05087 | acetyl-CoA acetyltransferase | 3.35 |
|  | FGSG_09321 | acetyl-CoA acetyltransferase IB | 0.44 |

^a^ Fold-change value represents the fold expression in *FgPTC3* deletion mutant ΔFgPtc3-8 as compared with that in the wild-type strain PH-1.
